# Multifunctional Roles of the Actin-Binding Protein Flightless I in Inflammation, Cancer and Wound Healing

**DOI:** 10.3389/fcell.2020.603508

**Published:** 2020-11-24

**Authors:** Xanthe L. Strudwick, Allison J. Cowin

**Affiliations:** Regenerative Medicine, Future Industries Institute, University of South Australia, Mawson Lakes, SA, Australia

**Keywords:** Flightless I, actin-binding protein, wound healing, inflammation, cancer

## Abstract

Flightless I is an actin-binding member of the gelsolin family of actin-remodeling proteins that inhibits actin polymerization but does not possess actin severing ability. Flightless I functions as a regulator of many cellular processes including proliferation, differentiation, apoptosis, and migration all of which are important for many physiological processes including wound repair, cancer progression and inflammation. More than simply facilitating cytoskeletal rearrangements, Flightless I has other important roles in the regulation of gene transcription within the nucleus where it interacts with nuclear hormone receptors to modulate cellular activities. In conjunction with key binding partners Leucine rich repeat in the Flightless I interaction proteins (LRRFIP)1/2, Flightless I acts both synergistically and competitively to regulate a wide range of cellular signaling including interacting with two of the most important inflammatory pathways, the NLRP3 inflammasome and the MyD88-TLR4 pathways. In this review we outline the current knowledge about this important cytoskeletal protein and describe its many functions across a range of health conditions and pathologies. We provide perspectives for future development of Flightless I as a potential target for clinical translation and insights into potential therapeutic approaches to manipulate Flightless I functions.

## Introduction

Flightless I (Flii) is an actin-binding protein that has been implicated in a wide range of biological processes, from those critical to ovulation and development, through to wound healing and cancer progression. Originally Flii was characterized in Drosophila, where mutations in the gelsolin domain caused disordered flight muscle myofibrils resulting in an inability to fly, as well as abnormal gastrulation during embryogenesis (Campbell et al., 1993; de Couet et al., 1995; Straub et al., 1996). Flii has since been shown to be a highly conserved protein in mammals with 95% homology observed between mouse and human and high expression in a wide range of human tissues with importance to mammalian health (Campbell et al., 1993, 1997; Nag et al., 2013). The complete loss of Flii has been shown to be embryonically lethal in mice, with failure of egg cylinder formation prior to gastrulation (Campbell et al., 2002) and human Flii maps within the critical region of chromosome 17 where contiguous-gene-deletion gives rise to the Smith-Magenis syndrome, the clinical features of which include short stature, brachydactyly, developmental delay, dysmorphic features, sleep disturbances, and behavioral problems (Chen et al., 1995). Flii has been found to have functions in many cellular processes that are importance for physiological processes including wound repair, cancer progression and inflammation. This review describes the current knowledge about this member of the gelsolin family of actin-remodeling proteins and describes its many important functions across a range of pathologies.

### The Actin Binding Protein Flii

**FIGURE 1:**
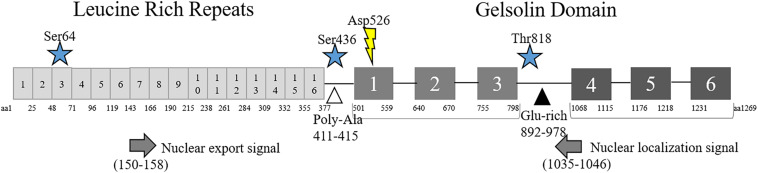
Structure of Flii. Schematic diagram of Flii protein including amino acid (aa) locations of the N-terminal Leucine Rich Repeats and C-terminal Gelsolin-like Domains, phosphorylation (star) and cleavage (lightning bolt) sites. Predicted nuclear localization (left arrow) and export (right arrow) signals and Poly-Ala (white triangle) and Glu-rich (black triangle) regions are also included.

Flii is a member of the gelsolin family of actin-binding proteins and consists of the classic 6-fold gelsolin repeat (GLD) at the C-terminal, in addition to 16 tandem leucine-rich repeats (LRR) at the N-terminus (Figure 1; Liu and Yin, 1998). Flii only has two of the six Ca^2+^ binding sites found in gelsolin that facilitate conformational change but is predicted to still exist in transition between a compact conformation and a more open form that can interact with actin (Nag et al., 2013). Flii binds actin via the gelsolin domain (Liu and Yin, 1998), localizing to actin-rich regions during embryogenesis (Davy et al., 2000) and with beta-tubulin- and actin-based structures at the periphery of cells that are stimulated to migrate (Davy et al., 2001). In mammalian cells, Flii binds both globular (G)- and filamentous (F)-actin, and has been shown to inhibit polymerization and cap the barbed end of F-actin, but it does not possess actin severing ability (Mohammad et al., 2012). More than simply being a member of the gelsolin family of actin-binding proteins, the additional N-terminal LRR marks Flii as unique amongst the gelsolin family, endowing it with the ability to interact with other proteins or lipids for molecular recognition (Liu and Yin, 1998). A number of binding partners have been revealed that interact with various domains within Flii, implicating roles in a wide range of cellular processes (summarized in Table 1). It may be that Flii effects are both cell specific, and dependent upon the availability of specific binding partners within each cell.

**TABLE 1:** Flightless Binding Partners.

Binding Partner	Flii Binding Domain	Function	References
**Actin Dynamics**			
Actin	GLD	Actin filament assembly and stabilization	Liu and Yin, 1998
G-Actin	GLD	Inhibit polymerisation	Mohammad et al., 2012
F-Actin	GLD	Caps barbed end	Mohammad et al., 2012
Ca^2+^	GLD (Glu1083) (Asp1194)	Type-2 Ca2+ binding sites control actinomyosin dynamics	Nag et al., 2013
Caspase-11	LLR and GLD	Localizes Caspase-11 to F-actin rich leading edge	Li et al., 2008
Daam1	GLD (Modules 4-6)	Disrupts Autoinhibition to enhance DRF-induced linear actin assembly	Higashi et al., 2010
mDia1	GLD (Modules 4-6)	Disrupts Autoinhibition to enhance DRF-induced linear actin assembly	Higashi et al., 2010
G3BP1	LRR	Adaptor protein to facilitate Rac1-mediated cell extension formation	Arora et al., 2018
IQGAP1	LRR	Facilitate interaction with cdc42 and R-Ras for cell extension and elongation	Arora et al., 2020
Kindlin-1	Not Defined	Binds this focal adhesion protein which links integrins to the F-actin cytoskeleton and regulates their activity	Kopecki et al., 2020
NMMIIA	GLD	Regulate formation of cell extensions and collagen compaction	Arora et al., 2015
Paxillin	Not Defined	Binds this focal contact protein which links integrin receptors to the actin cytoskeleton	Kopecki et al., 2009
P-Rex1	GLD	Rac1 effector to mediate RhoA-ROCK-independent myosin II activation and enhances collagen contraction	Marei et al., 2016
active Rac1	LRR	Rac1 effector to mediate RhoA-ROCK-independent myosin II activation	Marei et al., 2016
active R-Ras	LRR	Adaptor protein to facilitate Rac1-mediated cell extension formation	Arora et al., 2018
Ras Gap	LRR	Adaptor protein to facilitate Rac1-mediated cell extension formation	Arora et al., 2018
Robo-1	Not Defined	Association required for filopodial extensions on dendritic cells	Shrivastava et al., 2015
Talin	Not Defined	Binds this focal contact protein which links integrin receptors to the actin cytoskeleton	Kopecki et al., 2009
TGF-β1, 2, 3	Not Defined	Interacts with members of the TGFβ pathway in scratch wounded fibroblasts	Chan et al., 2014
Vinculin	Not Defined	Binds this focal contact protein which links integrin receptors to the actin cytoskeleton	Kopecki et al., 2009
**Transcription and Translation**			
AKT	Phosphorylation of Ser436	Recruits p62-associated cargoes to insoluble actin bundle portion, preventing p62 from recognizing LC3 and impeding autophagic clearance of ubiquitinated proteins within p62 cargoes	He et al., 2018
Androgen Receptor	LRR (aa1-494) and GLD (aa495-822)	Nuclear receptor coactivator to enhance transcription	Wang et al., 2016
BAF53	GLD (aa495-827)	Recruits the SWI/SNF ATP-dependent chromatin remodeling complex to the promotor region of ER target genes (transcriptional regulation)	Jeong et al., 2009
BRG1	Not Defined	Interaction of this subunit of the SWI/SNF complex and recruitment to the COL1A2 promoter region	Lim and Jeong, 2014
CaMK-II	Not Defined	Preferentially binds active CaMK-II to inhibit β-catenin dependent transcription	Seward et al., 2008
CARM1	LRR and GLD	Nuclear receptor coactivator to enhance transcription	Lee et al., 2004
ChREBP	LRR and GLD	Negative regulatory component of the ChREBP transcriptional complex	Wu et al., 2013
CISK phosphorylation	Phosphorylation of Ser436 and Thr818	Required for full function as ER co-activator	Xu et al., 2009
EEF2	Not Defined	Interacts with this essential factor for the translational process	Liao et al., 2015
Estrogen Receptor α	LRR GLD (G3 Module)	Hormone-independent transcriptional regulation Recruits SWI/SNF chromatin remodeling complex to the promotor region ER target genes to enhance transcription	Gettemans et al., 2005), (Jeong et al., 2009
Glucocorticoid Receptor	LRR	Activates GR-mediated transcription	Jin et al., 2017
GRIP1	LRR	Nuclear receptor coactivator to enhance transcription	Lee et al., 2004
Importin β	LRR	Interacts with this nuclear envelop associating protein involved in nuclear-cytoplasmic transport	Liao et al., 2015
LRRFIP1	LRR (aa1-427)	Interferes with LRRFIP1 to prevent β-catenin-dependent transcription	Seward et al., 2008)
Menin	Not Defined	Associates with this component of the MLL1/2 methyltransferase complex to facilitate chromatin recruitment and RNA Pol II residency for transcriptional regulation of SENP3-responsive homeobox genes	Nayak et al., 2017
MLL1/2	Not Defined	Associates with this component of the MLL1/2 methyltransferase complex to facilitate chromatin recruitment and RNA Pol II residency for transcriptional regulation of SENP3-responsive homeobox genes	Nayak et al., 2017
Nup88	LRR	Interacts with this nuclear pore complex protein involved in nuclear-cytoplasmic transport	Liao et al., 2015
p62	GLD Phospho-Ser436	Interferes with LC3-mediated p62-cargo engulfment by autophagosome	He et al., 2018
PPARγ	LRR (LXXLL Motif)	Prevents PPARγ receptor repression of the transcriptional activity	Choi et al., 2015
RbBP5	GLD (G1-3 Module)	Associates with this component of the MLL1/2 methyltransferase complex to facilitate chromatin recruitment and RNA Pol II residency for transcriptional regulation of SENP3-responsive homeobox genes	Nayak et al., 2017
SENP3	GLD (G1-3 Module)	Transcriptional regulation of SENP3-responsive homeobox genes. Recruits SENP3 to the promotor regions, facilitating mutual association with chromatin	Nayak et al., 2017
SMAD3	Not Defined	TGFβ-dependent interaction of this subunit of the SWI/SNF complex	Lim and Jeong, 2014
SNF2L	Not Defined	Transcriptional regulation via ISWI chromatin-remodeling complex	Pepin et al., 2013
Syncrip	Not Defined	Interacts with this RNA-binding protein involved in RNA metabolism, such as RNA stability, splicing, and translational control	Liao et al., 2015
Thyroid Receptor	LRR	Hormone-independent transcriptional regulation	Gettemans et al., 2005
UCP1	N/A	Cooperates with LRRFIP1 as transcriptional activator of UCP1 in brown adipose tissue thermogenisis	Shamsi et al., 2020
Ulk1	Phosphorylation of Ser64	Inhibits the phosphorylation by Akt, allowing autophagic clearance of ubiquitinated proteins within p62 cargoes	He et al., 2018
WDR5	Not Defined	Associates with this component of the MLL1/2 methyltransferase complex to facilitate chromatin recruitment and RNA Pol II residency for transcriptional regulation of SENP3-responsive homeobox genes	Nayak et al., 2017
**Inflammation**			
BCAP	Not Defined	Binding promotes Flii inhibition of NLRP3 Inflammasome activity	Carpentier et al., 2019
Caspase-1	LRR and GLD	Inhibits inflammatory activity and limit caspase-1 induced cell death	Li et al., 2008
Caspase-11	LRR and GLD	Directs localisation of Caspase-11 to F-actin rich leading edge and reduce TLR4 inflammatory signaling pathway	Li et al., 2008
LPS	LRR	Binds LPS to inhibit activation of macrophages	Lei et al., 2012
LRRFIP1	LRR (aa1-427)	Disrupts LRRFIP1-MyD88 binding to reduce TLR4 signaling	Dai et al., 2009
LRRFIP2	LRR (aa1-427)	Disrupts LRRFIP2-MyD88 binding to reduce TLR4 signaling Flii-LRRFIP2 binding enhances the interaction of Flii and Caspase-1 to inhibit NLRP3 Inflammasome	Dai et al., 2009) (Jin et al., 2013
MyD88	GLD	Interferes with formation of TLR4-MyD88 inflammatory signaling complex	Wang et al., 2006
NRX	Not Defined	Binding links Flii to MyD88 and synergistically prevent TLR4 inflammatory signaling pathway	Hayashi et al., 2010
RdCVF	Not Defined	Binding links Flii to MyD88 and synergistically prevent TLR4 inflammatory signaling pathway	Hayashi et al., 2010

**FIGURE 2:**
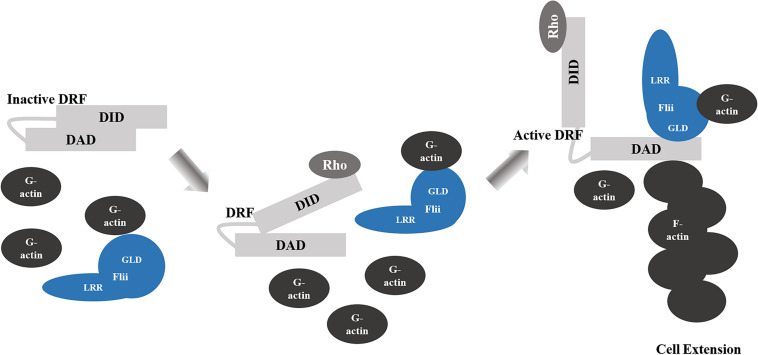
Flii Regulation of DRF-Mediated Actin Assembly. Binding of the diaphanous autoinhibitory domain (DAD) to the diaphanous inhibitory domain (DID) elements of Diaphanous-related formins (DRF) results in autoinhibition of the functional domains. Rho binding partially disrupts the DID-DAD interaction. The dissociation of the DID-DAD interaction is enhanced by Flii GLD bound to the DAD segment, which allows for activation of the DRF and filamentous actin (F-actin) assembly and the formation of cell extensions.

Flii is involved in the assembly of actin into filaments and is required for Diaphanous-related formins (DRF)-induced actin assembly (Figure 2; Higashi et al., 2010). Binding directly to DRFs, Disheveled associated activator of morphogenesis 1 (Daam1) and mammalian Diaphanous homolog 1 (mDia1) at the diaphanous autoinhibitory domain (DAD) Segment in the Carboxyl-terminal Region through its gelsolin 4–6 region, Flii enhances the intrinsic ability of DRF FH1-FH2 domains to assemble linear actin filaments (Higashi et al., 2010). Flii also promotes Rho-induced DRF actin assembly, by competing with the binding of the DRF amino-terminal diaphanous inhibitory domain (DID) domain to the DAD segment in the presence of active GTP-bound Rho, further disrupting intramolecular auto-inhibition that would normally restrain the actin assembly activity of DRF (Higashi et al., 2010). The transmission of HIV from dendritic cells to T-cells through cell-cell contact, relies upon Flii for sufficient formation of filopodial extensions by another DRF, Diaphanous 2 (Diaph2) (Shrivastava et al., 2015). When Flii is induced to bind with Roundabout 1 receptor (Robo-1), its ability to promote Diaph2 filopodia formation is perturbed and transmission of virus is reduced (Shrivastava et al., 2015). It is speculated that Flii may further support DRF actin assembly by holding the preformed F-actin or by recruiting globular actin (Goshima et al., 1999; Higashi et al., 2010). Thus, it appears that Flii may affect cellular migration by regulating the rearrangement of the actin skeleton. Indeed, Flii has been reported to have an inhibitory effect upon migration in skin fibroblasts, keratinocytes and bronchial epithelial cells (Cowin et al., 2007; Kopecki et al., 2009; Mohammad et al., 2012). Skin fibroblasts and keratinocytes isolated from mice with reduced Flii expression migrate faster following scratch wounding *in vitro*, whilst scratch wound closure is significantly delayed in cells from Flii overexpressing mice (Cowin et al., 2007; Kopecki et al., 2009). Similarly, Flii knockdown in human and mouse fibroblasts, or overexpression in mouse fibroblasts results in increased and decreased migration, respectively (Cowin et al., 2007; Mohammad et al., 2012). An inhibitory effect of Flii has also been reported in human bronchial epithelial cells, where Flii knockdown stimulates migration through transwells *in vitro*, whereas Flii overexpression inhibits this ability (Wang et al., 2017). In contrast, Marei et al., reported that in both NIH3T3 mouse embryonic fibroblasts and CHL1 human melanoma cells, Flii knockdown using two different siRNAs results in a decrease in the ability of cells to migrate in an Oris cell exclusion migration assay (Marei et al., 2016). In this study, loss of Flii was also associated with a decrease in accumulated distance, cell displacement and speed (but not directionality) using single cell tracking following scratch wounding (Marei et al., 2016). Likewise, siRNA knockdown of Flii in cells isolated from tendons inhibits scratch wound closure (Jackson et al., 2020b). The contradictory actions of Flii upon migration in different cell types is clearly demonstrated by Jackson et al who show that while fibroblasts isolated from mice with reduced levels of Flii show enhanced migration, tenocytes isolated from these same mice have significantly inhibited migration, and overexpression of Flii enhances their migration (Jackson et al., 2020a).

### Flii, Focal Adhesions and Cell Migration

Cell migration is tightly regulated by Rho GTPases, particularly Rho, Rac and Cdc42, which direct the polymerization, and depolymerisation of actin to dynamically rearrange the cytoskeleton to facilitate movement (Ridley, 2015). Of particular importance is the efficient turnover of adhesion sites to facilitate movement (Bach et al., 2009). Integrin receptor signaling initiates actin polymerisation and the formation of short lived cell-matrix adhesions termed focal complexes, localized under lamellipodia (Berrier and Yamada, 2007). A proportion of these focal complexes will develop into elongated focal adhesions, which are associated with contractile stress fibers and provide the force required to facilitate locomotion of the cell (Berrier and Yamada, 2007; Bach et al., 2009). A number of these focal adhesion can in turn transform into fibrillary adhesions that interact with the extracellular matrix to modify its structure and rigidity (Arnaout et al., 2007). Regulation of focal adhesion protein phosphorylation, such as paxillin by protein kinases including Src, dictates the regulation of focal adhesion turnover (Goetz, 2009; Huveneers and Danen, 2009).

Ras is a member of the Rho family of small GTPases which upon activation by exchange of GDP for GTP, in turn activates Phosphoinositide 3-Kinases (PI3K) to couple extracellular signals to actin polymerization via Rac1 (Olson and Marais, 2000). Cytoskeletal regulatory proteins containing LRR sequences similar to the Flii LRR, such as Rsp-1, were known to regulate Ras signal transduction and as such Flii was also predicted to regulate Ras signaling (Claudianos and Campbell, 1995). Flii was subsequently found to interact with the proline-rich sites of active R-Ras (but not K-Ras, H-Ras, or N-Ras) in mouse cells (Arora et al., 2018) as well as within focal adhesion fractions (Mohammad et al., 2012). It appears that Flii plays a central role in facilitating cell extension in the early phases of migration, by recruiting R-Ras to adhesion sites in spreading cells and acting as an adaptor protein to bring together R-Ras and GTPase-activating protein SH3 domain-binding protein (G3BP1) (Arora et al., 2018). The LRR of Flii binds R-Ras, the Ras GTPase activating protein (Ras GAP) and the C-terminus of G3BP1, which in turn can bind R-Ras via its C-terminus and via its N-terminus to Ras GAP, activating Ras to induce Rac1-mediated cell extension formation (Arora et al., 2018).

**FIGURE 3:**
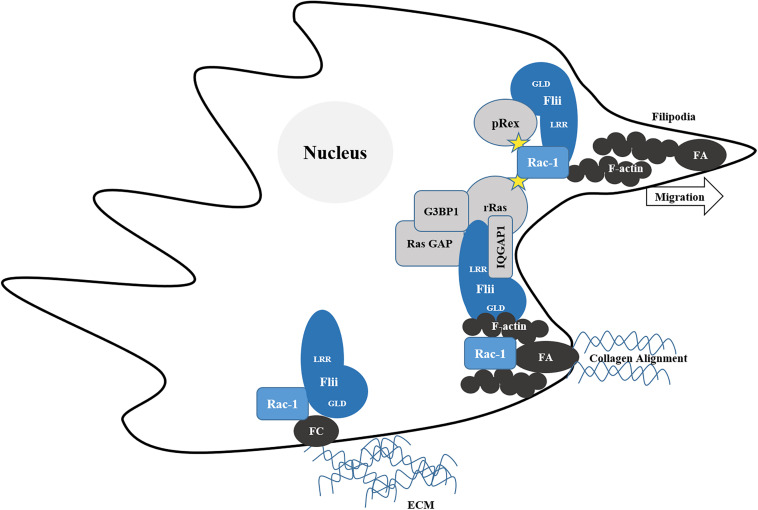
Flii regulates Rac-1 mediated migration. Migration relies upon the maturation of short-lived cell-extra cellular matrix (ECM) adhesions termed focal complexes (FC) into elongated focal adhesions (FA), associated with contractile stress fibers of filamentous actin (F-actin) which provide the force required align collagen and facilitate locomotion of the cell. Flii recruits R-Ras adhesion sites. The LRR of Flii binds R-Ras, the Ras GTPase activating protein (Ras GAP) and the C-terminus of G3BP1, which in turn can binds R-Ras via its C-terminus and via its N-terminus to Ras GAP, activating Ras to induce Rac1-mediated cell extension formation. Flii also activates Rac-1 via pRex to form filopodia and facilitate migration.

Rac1 is a primary mediator of the assembly of focal complexes at the leading edge of cells to facilitate lamellipodia formation and cellular migration (Rottner et al., 1999). Depending upon the specific guanine nucleotide exchange factor (GEF) which activates Rac1, different actin cytoskeletal arrangements are made which give rise to contrasting migratory phenotypes. Activation by GEP Tiam1 results in an anti-migratory phenotype with increased actin localization at cell–cell contacts, membrane ruffling and aggregation of NIH3T3 cells, whereas migration is dependent upon GEF P-Rex1 activation with cells exhibiting an elongated morphology and the formation of thin membrane protrusions rich in polymerized actin (Marei et al., 2016). In migrating CHL1 cells both P-Rex1 and Flii co-localize at the leading edge together with actin (Marei et al., 2016). Flii in fact binds preferentially to active Rac1 in human embryonic kidney (HEK293T) cells and also to its activator P-Rex1 (Marei et al., 2016). Migration occurs when myosin, an ATPase motor protein moves along actin filaments to translate chemical energy from ATP into mechanical force (Lodish et al., 2000). The binding of the Flii LRR to Rac1 is enhanced by P-Rex binding to Flii GLD, which in turn increases the phosphorylation of myosin light chain (pMLC) and activation of myosin II to mediate cell contractility and migration (Marei et al., 2016). Thus, Flii can act as a Rac1 effector (Figure 3) to mediate RhoA-ROCK-independent myosin II activation and stimulate migration (Marei et al., 2016).

While Flii plays a very clear role in facilitating focal adhesion formation, it does not appear that more Flii will necessarily equate to more migration. Indeed, adhesion is impaired in both fibroblasts and keratinocytes isolated from Flii overexpressing mice grown on fibronectin, laminin and collagen I and fibroblast morphology is altered with impaired spreading and reduced filopodia-like processes (Kopecki et al., 2009; Arora et al., 2015). Instead of forming focal adhesions only at the leading edge of motile cells, Flii overexpressing fibroblasts exhibit significantly increased levels of total F-actin, with increased numbers and size of focal adhesions and more prominent ventral stress fiber formation linking the adhesion sites across the periphery (Kopecki et al., 2011b). Furthermore, these adhesion sites appear to be converted into more stable focal complexes, which do not appear to readily turn over, due to reduced paxillin phosphorylation and increased α-actinin expression (Kopecki et al., 2011b). While no difference in the levels of active RhoA is observed in Flii overexpressing fibroblasts, there is a significant reduction in the levels of activated Rac1 and Cdc42 (Kopecki et al., 2011b). Mohammad et al further found that Flii knockdown results in the reduced formation of focal adhesions containing vinculin and activated β1 integrins, but elevated incorporation of G-actin into nascent filaments at focal adhesions (Mohammad et al., 2012).

While Flii does not directly bind integrin β1, β4, or hemidesmosome component tetraspanin CD151 in scratch wounded keratinocytes, it does bind focal contact proteins talin, paxillin, and vinculin, which are important proteins which link the integrin receptors to the actin cytoskeleton (Critchley et al., 1999; Kopecki et al., 2009). It appears that Flii may impact upon focal adhesion turnover, in part through its regulation of paxillin phosphorylation (Kopecki et al., 2011b). Flii overexpressing fibroblasts also have a significantly reduced ratio of activated p130Cas, with decreased expression of Src tyrosine kinase, both known to be involved in the Src mediated activation of Rac1 and Cdc42, phosphorylation of paxillin and subsequent membrane protrusion (Huveneers and Danen, 2009; Kopecki et al., 2011b). Taken together, these studies indicate that the effect of Flii upon migration is at least in part, due to its role in regulating focal adhesion maturation.

The maturation of focal adhesions into contractile fibrillary adhesions enables cells to modify the structure and rigidity of the surrounding extracellular matrix (Arnaout et al., 2007). At collagen adhesion sites, Flii associates with non-muscle myosin IIA (NMMIIA), a regulator of adhesion, polarity, and migration of non-muscle cells which is required for maturation of adhesions and the generation of contractile forces on collagen substrates (Alexandrova et al., 2008; Choi et al., 2008; Arora et al., 2015). The leading edge of extensions in cells spreading on collagen are enriched with TRPV4 channels leading to increased localized Ca^2+^ fluxes, which are required for the association of Flii with NMMIIA (Arora et al., 2015). Moreover, the LRR domain of Flii binds to Ras GTPase-activating-like protein (IQGAP1) to facilitate interaction with cdc42 and R-Ras to first form short cell extension (via cdc42), and then elongate these extensions (via R-Ras) responsible for collagen fibril compaction and alignment (Arora et al., 2020). Together with NMMIIA, Flii appears to promote the formation of cell extensions and collagen compaction (Arora et al., 2017). Flii-overexpressing fibroblasts form more elongated protrusions, penetrating further into the pores of collagen-coated membranes. The cells also remodel the surrounding collagen into more strongly compacted collagen fibrils, as well as displaying an increased uptake and degradation of exogenous collagen (Arora et al., 2015). Despite this apparent role in extracellular matrix remodeling, fibroblasts isolated from mice with altered levels of Flii do not exhibit differences in their ability to contract collagen gels *in vitro* (Kopecki et al., 2011b).

The process of cell migration is also dependent upon efficient disassembly of hemidesmosomes followed by the rapid formation of new and stable adhesions sites (Jones et al., 1998). Hemidesmosome formation is impaired in Flii overexpressing mice (Kopecki et al., 2009). In addition to an overall reduction in the number of hemidesmosomes, those present also have fewer sub-basal dense plates and shorter adhesion sites (Kopecki et al., 2009). Additionally, the basement membrane within the skin of Flii overexpressing mice have sparse tonofilaments and a decreased network of anchoring fibrils (Kopecki et al., 2009). Reducing Flii in heterozygous knockout mice, significantly increases CD151 and the basement membrane component laminin, as well as increases the level of integrin β4 chains in response to wounding (Kopecki et al., 2009). While integrin α6 is initially decreased in day 3 wounds, it is elevated during the later stages of healing in Flii heterozygous mice (Kopecki et al., 2009). The combination of integrin α6 and β4 is required for stable hemidesmosome formation (Dogic et al., 1998; Jones et al., 1998; Hintermann and Quaranta, 2004) and suggests that decreasing Flii expression can promote the stabilization of hemidesmosomes.

Distinct from the receptor-mediated pathways involving Rho, Rac and Cdc42, Flii also regulates migration through binding to the p30 domain of Caspase-11 via both its LRR and GLD where it localizes to the F-actin rich leading edge (Li et al., 2008). Its interaction with caspase-11 has no effect on caspase-11 activity (Li et al., 2008) which can regulate actin dynamics by actin depolymerisation to facilitate immune cell migration (Li et al., 2007). Caspase-11 binds to actin interacting protein 1 (Aip1), to promote the activation of cofilin by Aip1, and stimulates cofilin-mediated actin depolymerisation (Li et al., 2007). Moreover, Flii interacts with Ca^2+/^calmodulin (CaM)-dependent protein kinase type II (CaMK-II), which itself co-localizes with the actin cytoskeleton and influences cytoskeletal and focal adhesion dynamics to influence cell motility through dephosphorylation of focal adhesion kinase and paxillin (O’Leary et al., 2006; Easley et al., 2008). Regardless of the precise nature of the interaction it is clear that Flii acts as a key regulator of a number of pathways that influence cytoskeletal arrangement.

These cytoskeletal interactions exhibited by Flii are of critical importance in *C. elegans*, where Flii regulates the cytokinesis of somatic cells and appears essential for cell division, acting together with Ras to control the development of germline cells and interacts with the phosphoinositol-signaling pathway in the regulation of ovulation (Deng et al., 2007; Lu et al., 2008). Flii may also play a role in coordination of mammalian ovulation, as Flii has been shown to interact with the imitation switch (ISWI) ATPase homolog SNF2L that is expressed in mouse ovary granulosa cells (Pepin et al., 2013). SNF2L expression is required for normal follicle maturation and differentiation in luteal cells (Lazzaro et al., 2006) and appears to regulate fibrinogen-like 2 (Fgl2) expression in differentiating granulosa cells (Pepin et al., 2013). It may be that Flii interacts with SNF2L to regulate folliculogenesis in mammals and therefore play a role in ovulation in mammals. Whilst *C. elegans* Flii associates directly with Ras (Goshima et al., 1999), and colocalisation of Flii with both Ras and Rho has been observed at actin arcs, membrane ruffles and at the leading edge of motile mouse fibroblasts (Davy et al., 2001), the LRR of mammalian Flii does not directly bind to Ras or other small G proteins, such as Rac2, RhoA, or CDC42 in yeast-two hybrid or pull down assays (Liu and Yin, 1998). Nevertheless, Flii appears to play an important role in linking the structure of the cytoskeletal to transcriptional regulation (Lee et al., 2004).

**FIGURE 4:**
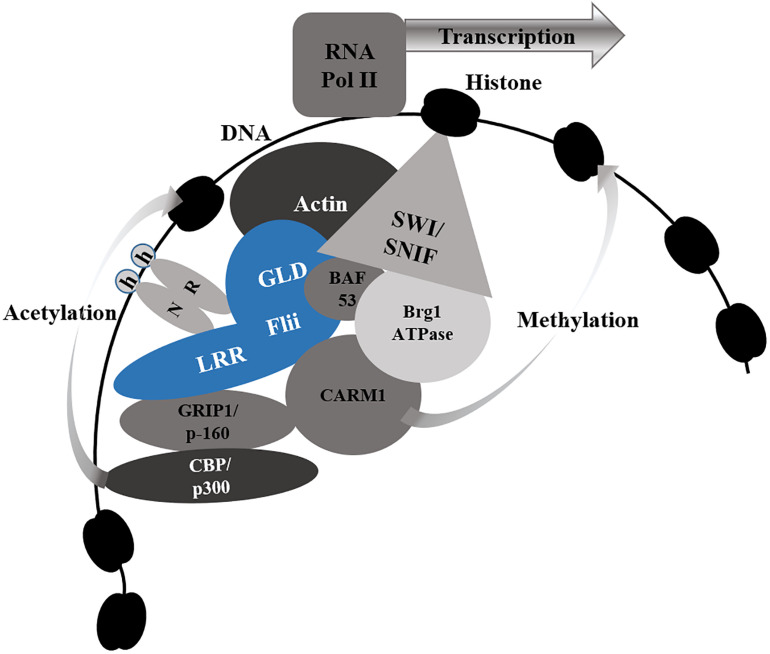
Flii as a Nuclear Receptor Co-Activator. Flii associates with nuclear receptors (NR) via the leucine rich repeat (LRR), the gelsolin domain (GLD) or both, in response to hormone (h) binding to NR. Flii (blue) facilitates chromatin remodeling by binding to key components of the SWI/SNF chromatin remodeling complex - actin and BAF53. It also brings together the p160 coactivator GRIP1 via the LRR domain and CARM1 via both the LRR and GLD to facilitate histone modification by acetylating CBP/p300 (bound to GRIP1) and methylating CARM1. Together these actions, allow Flii to enhance transcription by allowing for RNA Polymerase II (RNA Pol II) to bind to the exposed promotor region of the target genes.

Binding of Flii to the coactivator-associated arginine methyltransferase 1 (CARM1) occurs via both the N-terminal LRR domain and the C-terminal GLD (Lee et al., 2004). It also binds the p160 coactivator glucocorticoid receptor-interacting protein 1 (GRIP1- also known as steroid receptor coactivator-2 SRC-2) via the LRR domain (Archer et al., 2004; Lee et al., 2004). Flii and CARM1 act synergistically as secondary coactivators in the presence of the GRIP1/SRC-2 to facilitate histone modification and enhance ER mediated transcription (Lee et al., 2004). A schematic representation of the role of Flii as a NR co-activator can be found in Figure 4. Although not necessary for the interaction of Flii and ER coactivator GRIP1/SRC-2 complex, in order to function fully as an ER co-activator, Flii must first be phosphorylated at residues Ser436 and Thr818 by cytokine-independent survival kinase (CISK), a downstream effector of the PI 3-kinase, a pathway that is essential for the survival and proliferation of mammalian cells (Xu et al., 2009; Mendoza et al., 2011). Flii LRR binding to GR not only activates GR-mediated transcription (Jin et al., 2017), but also regulates GR occupancy at the promoter or the enhancer regions of ERα target genes, resulting in the loss of ERα from these regions in response to E2 and Dex treatment, thus, contributing to GR-mediated repression of ERα transcriptional activity (Yang and Jeong, 2019).

While Flii associates with the cytoskeleton and is co-localized with actin-based structures in motile mouse fibroblasts, it can translocate to the nucleus upon hormone stimulation or during periods of cell stress and gradually accumulates in the nucleus as cells approach confluence (Davy et al., 2001; Adams et al., 2008). While it is currently unclear whether the predicted nuclear localization (^1035^KRKFIIHRGKRK^1046^) and export (^150^LTDLLYLDL^158^) signals are functional (Gettemans et al., 2005), it is clear that Flii can be found both within the nucleus, or within the cytoplasm either associated the membrane/cystosol or the cytoskeleton (Davy et al., 2001). The translocation of Flii appears to be a gradual process when stimulated by serum starvation or where CaMK-II is inhibited. Under these conditions, nuclear accumulation of Flii appears to take 8–10 h, while nuclear export is somewhat faster, requiring 3-6 hours following CaMK-II activation (Seward et al., 2008). While Flii is predominantly observed within the cytosol of unwounded fibroblasts, within 30 mins of scratch wounding, it can be found within the nucleus and in the perinuclear region (Chan et al., 2014). It is interesting to note that translocation of Flii from the cytoplasm to the nucleus upon wounding is observed in fibroblasts, but not keratinocytes (Cowin et al., 2007), pointing to the complicated nature of Flii actions and its dependence upon specific cellular conditions.

### Flii and Transcriptional Regulation

More than simply facilitating cytoskeletal rearrangements, Flii has other important roles in the regulation of gene transcription within the nucleus. Flii interacts with nuclear hormone receptors including the androgen receptor (AR), estrogen receptor (ER), thyroid receptor (TR) and glucocorticoid receptor (GR) to regulate a number of cellular processes including proliferation, differentiation, apoptosis, and migration (Archer et al., 2004; Jin et al., 2017). Via its LRR, Flii associates with both ER and TR in a hormone-independent fashion (Gettemans et al., 2005). However, the expression of Flii in skin fibroblasts and keratinocytes is significantly enhanced with the addition of increasing concentrations of β-estradiol (Adams et al., 2008) and its occupancy of the promoter regions of ER target genes is increased in a hormone dependent manner (Lee et al., 2004; Jeong et al., 2009). Thus Flii binding to the ERα is enhanced in an estrogen-dependent manner, leading to enhanced transcription of estrogen responsive genes including trefoil factor family 1 (TFF1, also known as Ps2), growth regulation by estrogen in breast cancer 1 (GREB1), myelocytomatosis viral oncogene (Myc) and Cathepsin D (Jeong et al., 2009; Jeong, 2014). Flii can also bind the AR at the AR-ligand binding domain (residues 624–919) by both the LRR (residues 1–494) and residues 495–822 of the GLD, but not with residues 825–1268 (Wang et al., 2016). The recruitment of Flii to the promotor regions of target genes appears to be gene specific as, while Flii is required for TFF1/Ps2 expression, it is not required for ER-induced expression of the progesterone receptor (PgR), nor is it recruited to the promotor region of this gene (Won Jeong et al., 2012). Flii also forms a complex with AR in response to the AR ligand, competing with ligand binding to AR (Wang et al., 2016). In the presence of NCor and SMRT, known corepressors of AR, the interaction between Flii and AR is enhanced such that Flii inhibits AR transactivation which results in reduced AR nuclear localisation and repression of AR-dependent signaling (Wang et al., 2016).

Flii binds not only to actin in its globular and filamentous forms, but also to actin related proteins including Brg- or Brm-associated factor 53 (BAF53) (Goshima et al., 1999; Lee et al., 2004). Both actin and BAF53 are key components of the SWI/SNF chromatin remodeling complex, required for the initiation of transcription of nuclear receptor target genes. For transcription to occur, a SWI/SNF complex incorporating two molecules of actin, BAF53, or one of each must first be formed at the promoter site (Archer et al., 2005). Flii is required for the maintenance of optimal chromatin configuration at the enhancers of estrogen target genes, to facilitate binding of RNA polymerase II to the promotor region of the target gene (Jeong, 2014). In the case of ERα-mediated transcription, Flii binding to the ERα and BAF53 via its C-terminal GLD, recruits the SWI/SNF ATP-dependent chromatin remodeling complex to the promotor region of estrogen receptor target genes (Jeong et al., 2009). Flii in association with BAF53 is also required for the early recruitment of BRG1 to the promotor regions or estrogen responsive genes TFF1/pS2 and GREB1, ahead of ER recruitment and efficient expression of estrogen target genes (Jeong et al., 2009).

Flii regulates transcription of target genes downstream of other non-hormone dependent nuclear receptors, including those which regulate cell differentiation as well as glucose and lipid metabolism. Flii interacts with ISWI chromatin-remodeling complex through its association with SNF2L to regulate transcription of fibrinogen-like 2 (Fgl2) expression in differentiating granulosa cells (Pepin et al., 2013). Flii also acts independently of ERα-mediated gene regulation to facilitate chromatin recruitment and RNA Polymerase II residency to regulate transcription of SENP3-responsive homeobox genes, DLX3, HOXA9, HOXB3, MEIS1, and MEOX1 required for human osteogenic differentiation (Nayak et al., 2017). Here, Flii associates with both SENP3 and components of MLL1/2 methyltransferase complex including RbBP5, menin, WDR5 and the catalytic core subunits MLL1 and MLL2 (Nayak et al., 2017). The presence or absence of Flii does not affect the association of RbBP5, MLL1, or MLL2 with the DLX3 gene, rather Flii is required for the recruitment of SENP3 to the promotor region and exon 3 of DLX3 facilitating mutual association with chromatin (Nayak et al., 2017).

The dual action of Flii in facilitating the initiation of transcription through chromatin remodeling and co-activator recruitment positions Flii as a key regulator of nuclear-receptor transcription. However, it may be that the widespread impacts of Flii upon cellular processes may be due to the ability of Flii to regulate nuclear transport. Flii interacts with the nuclear envelop associating proteins Importin β and Nup88 via the LRR domain (Liao et al., 2015). Both of these proteins are involved in nucleocytoplasmic transport of mRNA, protein and the 60S ribosomal complex, Importin β as a nuclear transport receptor and Nup88 that is a component of the nuclear pore complex (Strom and Weis, 2001; Bernad et al., 2006; Hutten and Kehlenbach, 2006). Furthermore, over half of the 133 putative Flii binding partners identified by immunoprecipitation and LC-MS/MS analysis in the H1299 lung cancer cell line are associated with nucleocytoplasmic transport of both RNA and protein, post-translational modifications of RNA and the biosynthesis of protein (Wang et al., 2017). Flii knockdown and overexpression significantly affects both the nucleus/cytoplasm ratio of mRNA and the level of ribosome-nascent chain complex-associated mRNAs (Wang et al., 2017).

### Transcriptional Regulation and Metabolism

Flii has been shown to act as a repressor of the transcriptional activity of the Peroxisome Proliferator-Activated Receptor γ (PPARγ) and its overexpression suppresses adipogenesis (Choi et al., 2015). Flii is expressed more highly in adult bovine adipose tissue compared to fetal bovine adipose tissue (Zhou et al., 2014), with Flii expression increasing in differentiated adipocytes compared with preadipocytes (Liu et al., 2016). In the absence of ligand, the LXXLL motif within the LRR domain of Flii binds directly to the DNA binding domain of the receptor to prevent PPARγ receptor occupancy at the promotor of target genes as well as blocking the interaction between PPARγ and retinoid X receptor α (RXRα) (Choi et al., 2015). Flii was widely expressed in human tissues, with strongest expression in skeletal muscle (Campbell et al., 1997). Genetic characterisation of a number of Chinese cattle breeds, which historically exhibit small body size and low intramuscular fat content compared to other meat production breeds, shows that three polymorphisms in Flii are associated with increased body mass, height and length, as well as chest girth. Moreover, these polymorphisms; CT (rs41910826), TT (rs444484913), and CA (rs522737248) were associated with increased PPARγ in adult adipose as well as increased Flii in fetal muscle (Liu et al., 2016).

Recently, it has been shown that Drosophila with mutations in Flii are resistant to starvation, with increased triglyceride levels in body fat and intestine due to elevated desaturase 1 (desat1), whose preferred substrate is stearoyl-CoA (Park et al., 2018b). Conversely, overexpression of Flii reduced both the amount of triglycerides and the expression of desalt1, which was replicated in mammalian preadipocytes (Park et al., 2018b). Flii is also a transcriptional coactivator of Uncoupling protein-1 (UCP1), a key regulator of brown fat adipogenesis, and acts to modulate systemic energy metabolism (Shamsi et al., 2020). A number of other enzymes related to the metabolic pathways of glycolysis, lipogenesis, lypolysis and the pentose phosphate pathway are also increased in the Drosophila Flii mutants (Park et al., 2018a). Mutations in the Flii gene have been associated with increased insulin resistance, with a higher expression of most glycolytic-enzyme genes (Park et al., 2018a). It appears that Flii acts as a component of the glucose-responsive transcription factor carbohydrate responsive element binding protein (ChREBP) transcription complex, colocalising to interact with ChREBP and down regulate ChREBP-mediated transcription in colorectal cancer and hepatocellular carcinoma cells (Wu et al., 2013). These studies suggest that Flii may be an important regulator of metabolism and may highlight Flii as a therapeutic target for the management of obesity and metabolic diseases. Interestingly, Mediterranean fruit fly larvae that are fed on a fatty acid deficient diet exhibit markedly increased Flii expression and a significantly reduced flight ability (Cho et al., 2013). This raises the question as to whether dietary changes may impact upon Flii expression in mammals and if changing dietary fatty acids may reduce Flii expression to improve health outcomes.

### Flii and Its Binding Proteins LRRFIP1/2

Leucine rich repeat Flightless-interacting protein (LRRFIP)1/2 are key binding partners of Flii known to act both synergistically and competitively to regulate a wide range of signaling pathways. The LRR of Flii interacts with the double stranded RNA binding protein TAR RNA interacting protein (TRIP) (Wilson et al., 1998) which is also known as the short mouse homolog FLI LRR associated protein (FLAP-1), Leucine rich repeat in the Flightless1 interaction protein 1 (LRRFIP1) and GC-binding factor 2 (GCF2), as well as the closely related protein, Leucine rich repeat in the Flightless1 interaction protein (LRRFIP2) (Liu and Yin, 1998; Fong and de Couet, 1999). Herein, these will be referred to as LRRFIP1/2 for consistency.

Leucine rich repeat in the Flightless1 interaction protein 1 is a cytosolic nucleic-acid sensor, which mediates type I interferon (IFN) production, and acts a transcriptional repressor of EGFR, PDGF, TNFα and the glutamine transporter EAAT2 (Ohtsuka et al., 2011; Jin et al., 2013). While LRRFIP1 normally induces type I IFN expression in virally infected 3T3 cells, overexpression of LRRFIP1 can induce IFN expression regardless of infection status (Bagashev et al., 2010). LRRFIP1 rapidly colocalises with viruses and interacts transiently with viral sensing Toll-like receptor 3 (TLR3) following viral infection (Bagashev et al., 2010). As well as inducing IFN production in fibroblasts, LRRFIP1 also induces IFN expression in macrophages and hepatocytes (Yang et al., 2010; Liu et al., 2015). While viral infection with Hepatitis C (HCV) in the cells does not alter expression of LRRFIP1 itself, the induction of IFN by LRRFIP1 is also exacerbated by HCV infection, and the upregulation of IFN acts to inhibit the replication of the virus (Liu et al., 2015). Silencing LRRFIP1 can also affect inflammasome activation and IL-1β secretion (Jin et al., 2013). LRRFIP2 acts as a positive regulator of TLR4 signaling by competitively disrupting the interaction between MyD88 and Flii at a very early stage of TLR agonist stimulation (Jin et al., 2013), and interacts with downstream protein caspase-11 (Li et al., 2008).

LRRFIP1 is also required for non-canonical Wnt3A stimulated, PCP pathway activation of small GTPases, Rho, Rac and Cdc42 to direct cell migration, wherein LRRFIP1 localized within perinuclear regions binds the PDZ domain of Dvl3, that is active specifically in PCP pathway signaling (Ohtsuka et al., 2011). LRRFIP1 enhances vascular smooth muscle cells (VSMCs) proliferation and ERK phosphorylation, which, together with remodeling are important pathological events in atherosclerosis and restenosis. Reducing LRRFIP1 prevents neointimal hyperplasia in mouse carotid artery injury (Choe et al., 2013) and has also been identified as critical to platelet function, positively regulating thrombus formation and in human platelets interacts with Flii and the platelet cytoskeletal protein Drebrin 1(Goodall et al., 2010).

Moreover, both LRRFIP1 and LRRFIP2 act as important activators of the canonical β-catenin/TCF/LEF signaling pathway, binding to Wnt signal mediator Dishevelled (Dvl) as well as β-catenin, glucocorticoid receptor interacting protein 1 (GRIP1), and p300 (Lee et al., 2004; Liu et al., 2005; Yang et al., 2010; Ohtsuka et al., 2011), which leads to transcription of c-myc and cyclinD1, which stimulates proliferation and cell cycle progression as well as apoptosis (Lee and Stallcup, 2006; Liao et al., 2007). Flii disrupts the ability of LRRFIP1 and p300 to synergistically activate transcription by β-catenin and TCF/LEF and thus acts as a negative regulator of the canonical Wnt signaling pathway (Lee and Stallcup, 2006). The Wnt signaling pathway itself modulates the nuclear receptor pathways as β-catenin enhances AR-dependent transcription through direct interaction of β-catenin, Flap1, p300 and AR (Truica et al., 2000; Lee and Stallcup, 2006). Flii may act as the determining factor in maintaining the balance between NR and β-catenin/LEF1/TCF mediated transcription, dependent upon nuclear levels of Flii (Lee and Stallcup, 2006). The interaction of Flii with LRRFIP1/2 is of particular importance in the regulation of cell survival, particularly with regards to regulation of proliferative or apoptotic pathways.

### Flii and Cell Survival – Proliferation vs Apoptosis

Numerous studies have shown that Flii is involved in the regulation of proliferative and apoptotic pathways. Flii is generally described as a negative regulator of proliferation with siRNA knockdown of Flii in both fibroblasts and keratinocytes resulting in increased proliferation and cells isolated from Flii overexpressing mice displaying reduced proliferative ability (Cowin et al., 2007). Flii also negatively regulates the canonical Wnt signaling pathway through disrupting the binding of LRRFIP1/2 with β-Catenin (β-Cat) (Lee and Stallcup, 2006). The canonical Wnt-signaling pathway regulates the expression of proliferative genes by tightly controlling the phosphorylation and degradation of cytosolic β-Cat by the Axin complex, and the ability of β-Cat to act as a transcriptional co-activator within the nucleus (MacDonald et al., 2009). Cytosolic Flii acts upon β-Cat dependent cyclin D1 transcription and cell cycle progression in mouse fibroblasts, where it preferentially binds active Ca^2+/^calmodulin (CaM)-dependent protein kinase type II (CaMK-II) but is not phosphorylated by CaMK-II (Seward et al., 2008). Flii inhibition of cyclinD1 transcription occurs without β-Cat degradation. Instead it appears that when CaMK-II becomes inactive due to contact inhibition and a reduction in Ca^2+^ transients, Flii gradually relocates to the nucleus where it interferes with LRRFIP1 and LRRFIP2 to prevent β-Cat-dependent transcription of cyclin D1 and reduces proliferation (Seward et al., 2008). LRRFIP1 and LRRFIP2 also binds CaMK-II within the cytosol, which may further indicate that the regulation of proliferation is dictated by subtle changes in the ratio of Flii, β-Cat, LRRFIP1/2 and Tcf/Lef factors (Seward et al., 2008).

In contrast to the negative effect on proliferation described above, a positive proliferative response to Flii is observed within the germinal matrix of the claws of mice that constitutively overexpress the protein and claw regrowth is subsequently enhanced in these mice (Strudwick et al., 2017). The germinal matrix is the organ that supplies the pool of keratinocytes that undergo proliferation and differentiation to form the nail or claw of the digit tip (De Berker et al., 2000; Strudwick et al., 2017). In this case, β-Cat expression is maintained, with continued expression of cyclin D1 within the germinal matrix of the regenerating claws in these Flii overexpressing mice after proximal amputation (Strudwick et al., 2017). Likewise, proliferation is enhanced in tenocytes isolated from Flii overexpressing mice (Jackson et al., 2020a) and siRNA knockdown reduces proliferation of these cells. It was also observed that increasing the level of Flii in an injured tendon resulted in reduced tendon adhesion formation and better healing outcomes (Jackson et al., 2020b). A similar positive role for Flii is seen in the MCF-7 breast cancer cell line (which contain the estrogen-inducible TFF1/pS2 gene), where silencing Flii results in significantly inhibited proliferation (Jeong, 2014). This may be due to the Flii co-activator function of the ER being reduced. Indeed, CISK phosphorylated Flii is required for E2-dependent cell growth (Xu et al., 2009). Moreover, CISK and Flii are seen to promote cell survival in 32D cells, protecting them from IL-3 withdrawal-induced apoptosis (Xu et al., 2009). Here, the CISK-phosphorylated Flii, enhances ER activity (Xu et al., 2009), which is also likely to be at play in other cell types in which the ER is active, such as MCF-7 cells, where estrogen is known to protect MCF-7 cells from apoptosis (Wang and Phang, 1995). Flii further inhibits apoptosis, through its interaction with caspase-1. Flii binds caspase-1 via both the LRR and GLD to inhibit its activity and limit caspase-1 induced cell death (Li et al., 2008). However, Flii does not inhibit caspase-11–induced cell death in HeLa cells (Li et al., 2008). In fact, the interaction of Flii with caspases may play a more important role in the regulation of inflammation, than in simply promoting cell survival.

### Flii and the Immune Response

**FIGURE 5:**
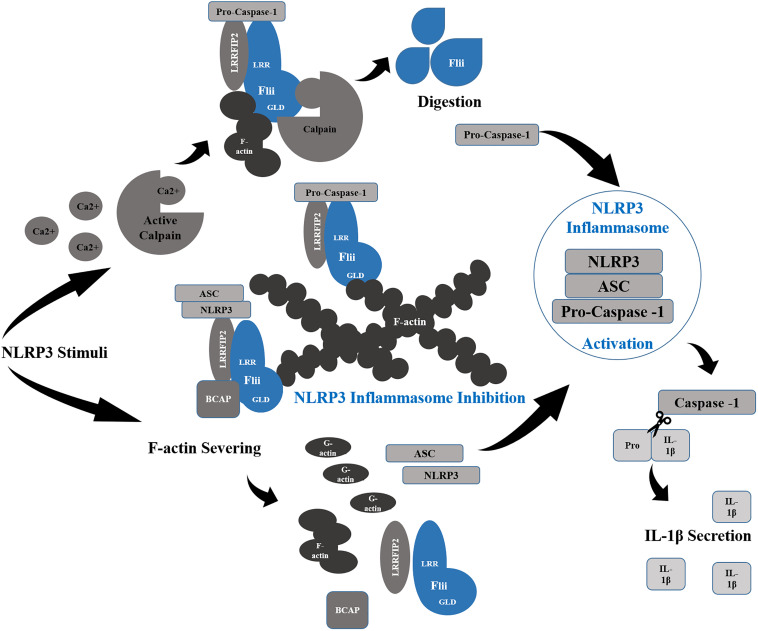
Flii and NLRP3 Inflammasome Regulation. Flii works synergistically with LRRFIP2 to inhibit the NLRP3 Inflammasome through its interaction with pro-Caspase-1, sequestering it to filamentous actin (F-actin). Moreover, Binding to LRRFIP2 in the presence of BCAP, Flii strengthens the inhibitory effect of LRRFIP2 upon the inflammasome, sequestering NLRP3 and ASC also to F-actin rich sites. In order for the activation of the inflammasome to occur, Ca2+ released following NLRP3 stimuli activates Calpain to digest Flii and release Pro-Caspase-1. Actin severing into G-actin also disrupts in association of Flii and LRRFIP2 with NLRP3 and ASC, allowing the formation of the NLRP3 inflammasome and the activation of Caspase-1 which cleaves Pro-IL-1β into its active form for secretion.

Flii plays an important role in the regulation of innate immunity which appears to be conserved from invertebrates through to humans, interacting with two of the most important inflammatory pathways, the NLRP3 inflammasome (Figure 5) and the MyD88-TLR4 signaling pathway (Wang et al., 2006; Zhang et al., 2015). Upon priming with bacterial LPS and ATP stimulation, the NLRP3 inflammasome activates pro-caspase 1, stimulating pyroptosis of macrophages to enhance bacterial clearance (Miao et al., 2011). Cleavage of IL-1β also occurs for secretion and further pro-inflammatory activity (Mangan et al., 2018). Activation by ATP or nigericin also induces translocation of NLRP3 inflammasome components, NLRP3 and ASC, whereupon they interact with F-actin microfilaments that dampens activity of the inflammasome (Burger et al., 2016). In resting macrophages, Flii and LRRFIP2 are co-localized with F-actin and it is the recruitment of the NLRP3 inflammasome upon stimulation that allows for the inhibitory action of Flii to occur (Burger et al., 2016). Silencing either Flii or LRRFIP2 abrogates the colocalization of NLRP3 with F-actin (Burger et al., 2016) indicating that they are critical for stabilizing this interaction. Knockdown of Flii enhances NLRP3 inflammasome activation (Jin et al., 2013) and overexpression *in vitro* reduces IL-1β maturation and secretion suggesting that Flii can act as a negative regulator of the NLRP3 inflammasome (Li et al., 2008; Jin et al., 2013). Furthermore, the close interaction of Flii with the NLRP3 inflammasome enables the dampening of its activity. The binding of Flii to pro-caspase-1 to prevent the formation of the NLRP3 inflammasome is enhanced by the interaction of Flii and LRRFIP2 with B cell adaptor for phosphoinositide 3-kinase (PI3K) (BCAP) (Carpentier et al., 2019). The inhibitory effect of Flii is further enhanced by LRRFIP2 binding both NLRP3 by its N terminal and Flii by its coiled motif to enhance the interaction of Flii and Caspase-1 (Jin et al., 2013). Silencing LRRFIP2 in macrophages results in greater NLRP3 inflammasome activation, increased cleaved Caspase-1 and increased IL-1β secretion following LPS priming and ATP stimulation (Jin et al., 2013).

Flii binds Caspase-1 acting both as a pseudo substrate of Caspase-1 and a potent inhibitor of IL-1β maturation and secretion (Li et al., 2008). Caspase-1 cleaves Flii into three C-terminal fragments, one around 90 kD and one around 60 kD as well as the 100-kD fragment (Li et al., 2008). Although only one potential cleavage site Asp526 and one recognition site ^523^YEADC^527^ localized within the GLD was for found caspase-1 and no site for any other caspases, Caspase-11 also cleaves Flii into a C-terminal cleavage fragment around 100 kD (Li et al., 2008). The cleavage of Flii is not required for it to act as an inhibitor (Li et al., 2008) and the function of the cleaved forms of Flii have yet to be elucidated. Zhang et al. have found that, in canonical NLRP inflammasome activation, calpain activity is essential for releasing caspase-1 from Flii and the cytoskeleton, allowing its function in IL-1β maturation (Zhang et al., 2015). Flii may not only prevent the inflammatory action of Caspase-1, but direct it towards its actin remodeling role, as the co-expression of Flii with caspase-1 in COS cells enriches Caspase-1 localisation at the leading edge of motile cells (Li et al., 2008). Similarly, Flii recruits Caspase-11 to the Triton X-100 insoluble actin bundle fraction to reduce inflammasome activation (Li et al., 2008). The interaction between caspase-11, LRRFIP2 and Flii is also likely to regulate the immune response through influencing the ability of caspase-11 to facilitate immune cell migration and promote bacterial clearance via phagosome fusion with lysosomes (Li et al., 2007; Akhter et al., 2012). Moreover, Flii interaction with the MyD88-TLR4 signaling pathway acts to regulate the early burst of inflammation in response to injury and pathogen recognition (Wang et al., 2005; Mogensen, 2009).

TLRs signal through MyD88 or TRIF and activate NFκB, MAP kinases, and IRF molecules (Bagashev et al., 2010). *In vitro*, Flii binds MyD88 and interferes with the formation of TLR4-MyD88 signaling complex to inhibit LPS induced NFκB activation in macrophages (Wang et al., 2005). LRRFIP1 also interacts with Flii and MyD88 and both LRRFIP1 and 2 positively regulate TLR signalling (Dai et al., 2009). In this setting Flii disrupts the binding of LRRFIP1/2 with MyD88 to negatively regulate TLR4 signaling (Dai et al., 2009). Decreasing Flii expression results in increased TNFα and IL-8 in response to IL-1 and LPS treatment, whilst its overexpression significantly inhibits LPS or lipid A-induced NF-κB activation and blocks IL-1- and LPS-induced IL-8 promoter activity (Wang et al., 2006). Flii overexpression does not affect TNF-α-induced IL-8 promoter activity that is MyD88-independent (Wang et al., 2006), indicating a specific role for Flii in the MyD88/TLR4 pathway.

Cells stimulated with LPS in the presence of culture media enriched with Flii also show reduced production and secretion of TNFα (Lei et al., 2012) suggesting that secreted Flii plays a role in this pathway. Flii is found within the plasma of healthy volunteers (Lei et al., 2012) and its secretion is increased in response to wounding (Cowin et al., 2007; Ruzehaji et al., 2012). Extracellular Flii is found in wound fluids collected from blisters, acute and chronic wounds (Cowin et al., 2012; Ruzehaji et al., 2012). Flii is constitutively secreted through a non-classical late endosome/lysosome-mediated pathway by both fibroblasts and macrophages, and its secretion is upregulated both in response to scratch-wounding in fibroblasts or following lipopolysaccharide (LPS) activation of macrophages (Cowin et al., 2007; Cowin et al., 2012; Lei et al., 2012). Secreted Flii can bind LPS with its N-terminal LRR (Lei et al., 2012), which has high sequence homology to TLR4, known to play a key role in detecting bacteria (Bell et al., 2003). It appears that secreted Flii may sequester LPS, preventing the activation of macrophages to reduce cytokine production (Lei et al., 2012).

Inside the cell, Flii is located in the MyD88/TLR4 complex through its interaction with nucleoredoxin (NRX) (Hayashi et al., 2010). NRX, like Flii is a negative regulator of the Wnt signaling pathway through Dishevelled (Dvl), where it interacts with the basic-PDZ domain of Dvl (which functions in non-canonical PCP pathway (Ohtsuka et al., 2011)) in a redox-dependent manner and mediates the redox-dependent activation of the Wnt/β-catenin pathway (Funato et al., 2006). NRX and its subfamily members (RdCVF and C9orf121, but not TRX) may have a common role through their interaction with Flii in TLR4/MyD88 signaling pathway (Hayashi et al., 2010). The ability of NRX and Flii to form a ternary complex with actin is disrupted by ethanol contributing to the progression of alcoholic liver disease in mice, which is also characterized by altered MyD88/TLR4 expression (Alarcon-Sanchez et al., 2020). Both RdCVF and NRX link Flii to MyD88, and synergistically prevent LPS-induced MyD88/TLR4 NFκB activation (Hayashi et al., 2010). However, as Dvl binds specifically to NRX, but not other redox-regulating family members, it seems that NRX alone plays a dual role with Flii in both the Wnt signaling pathway and the TLR4/MyD88 pathway (Hayashi et al., 2010). Flii expression is increased following macrophage stimulation with LPS for 12 hours (Jin et al., 2013). Moreover, as overexpression of Flii does not change MyD88 expression, nor is Flii expression induced upon shorter LPS stimulation it is not yet known how Flii serves as an inhibitor of TLR signaling in shorter term responses (Wang et al., 2006). It appears that release of Flii from one signaling pathway, to allow for its action in alternate processes may be triggered upon stimulation and further investigation of the kinetics and conditions required for specific Flii actions would clarify this issue.

### Flii Regulation of the TGFβ/Smad-Dependent Signaling Pathway

TGF-β1 (along with TGF-β2) regulates collagen production and plays a major role in fibrosis and scar formation following tissue injury, while TGF-β3 is anti-fibrotic and stimulates epidermal and dermal cell migration (Biernacka et al., 2011; Huang et al., 2014). Signaling via Smad3 or Akt, TGF-β1 stimulates collagen I transcription and the activation of fibroblasts into contractile, pro-fibrotic myofibroblasts (Biernacka et al., 2011). Flii co-precipitates with TGF-β1, TGF-β2, TGF-β3 and Akt isolated from the nucleus of scratch wounded fibroblasts, as well as it co-localizes with Smad 2/3 and 7 in both the nucleus and cytoplasm of these same cells (Chan et al., 2014). Reducing Flii by siRNA in human foreskin fibroblasts significantly reduces TGF-β1 expression (Adams et al., 2008). Fibroblasts isolated from Flii overexpressing and Flii deficient mice, reveals that while TGF-β1 mRNA is increased in Flii overexpressing fibroblasts, and decreased in Flii deficient cells, no change in TGF-β3 mRNA is observed (Chan et al., 2014).

Flii appears to play gender specific roles in TGF-β1 regulation as male Flii overexpressing, but not female mice, display up-regulated TGF-β1 and this is most pronounced in aged male Flii overexpressing wounds (Adams et al., 2008). Nevertheless *in vitro*, when Flii expression is reduced by siRNA, Smad 3 gene expression is also reduced, while the inhibitory Smad 7 (which competes with Smad 3 for receptor interaction and marks them for degradation (Moustakas et al., 2001)) is upregulated (Chan et al., 2014). Thus, Flii upregulates TGF-β1 signaling and increased Flii expression is associated with dose dependent increases in type 1 collagen (COL1A2) expression in A549 cells (Lim and Jeong, 2014). While changing the levels of GRIP1, CARM1 or p300 does not result in synergistic activation of COL1A2, it appears that Flii acts by recruiting BRG1 to promotor region of COL1A2 increasing chromatin accessibility at the COL1A2 promotor carried out by SWI/SNF complex (Lim and Jeong, 2014). Collagen I expression and secretion is also reduced in fibroblasts treated with Flii siRNA (Cowin et al., 2007) and siRNA knockdown of Flii inhibits estrogen-mediated collagen I secretion by fibroblasts *in vitro* indicating that Flii is required for collagen I production (Adams et al., 2008). Elevated Flii in primary fibroblasts isolated from mice with the skin blistering disorder Epidermolysis Bullosa Acquisita (EBA) impairs collagen contraction, however, altering Flii levels in normal mice does not affect the contractile ability of fibroblasts *in vitro* (Kopecki et al., 2011a). It is interesting to note that exogenous addition of TGF-β1 is able to restore the contractile ability of fibroblasts isolated from Flii overexpressing EBA mice (Kopecki et al., 2011a). As expression of P-Rex1 with which Flii interacts, but not Tiam1 in primary human fibroblasts, enhances fibroblast-collagen matrix contraction, increases collagen content and crosslinking and significantly increased pMLC (Marei et al., 2016), it may be that the increased contractility and collagen deposition observed in pathological Flii overexpressing fibroblasts may be via selective pathway activation under differing conditions, in this case which may be through its enhancement of P-Rex1 activation of Rac1. Clearly, Flii can play both positive and negative roles in cell recruitment and migration, immune response stimulation and resolution, proliferation and apoptosis, extra cellular matrix deposition and remodeling, all of which point to the critical importance of Flii as a key regulator of many physiological processes.

### Flii as a Negative Regulator of Wound Healing

Fetal wounds heal without scar formation via the purse-string closure of actin-myosin cables,(Martin, 1997; Cowin et al., 2003). However, a switch to a more adult-type, scar-forming healing response reliant upon lammellipodial crawling of epidermal cells upon a provisional wound matrix occurs around the start of the third trimester (embryonic day 18 in rats) (Martin, 1997). The expression of Flii in the developing skin increases dramatically around the time of this switch while it is noticeably absent in keratinocytes surrounding the wound of early gestation wounds (Lin et al., 2011). Moreover, while Flii does not co-localize with actin-myosin cables formed around E17 wounds, it is found highly expressed within keratinocytes at the leading edge of E19 wounded explants (Lin et al., 2011). This suggests that Flii plays an important role in the cytoskeletal mechanics required for cell migration during wound healing. Flii expression is relatively low in unwounded adult skin but rapidly increases in response to wounding (Ruzehaji et al., 2014). Despite being induced by wound healing, Flii play a generally negative role such that reducing Flii by heterozygous knockout improves healing rates with lower collagen I deposition and overexpression of Flii leads to impaired healing with evidence of scar formation and increased collagen I deposition (Cowin et al., 2007). Moreover, the negative impact of Flii upon wound healing may also be due to its inhibition of epidermal stem cell activation (Yang et al., 2020). These cells, that reside within hair follicles adjacent to the wound edge, require activation in order to produce proliferative progeny which contribute to re-epithelisation of the wound (Plikus et al., 2012). However, high levels of Flii appear to interrupt the Wnt-signaling pathway responsible and thus delayed wound closure (Yang et al., 2020).

**FIGURE 6:**
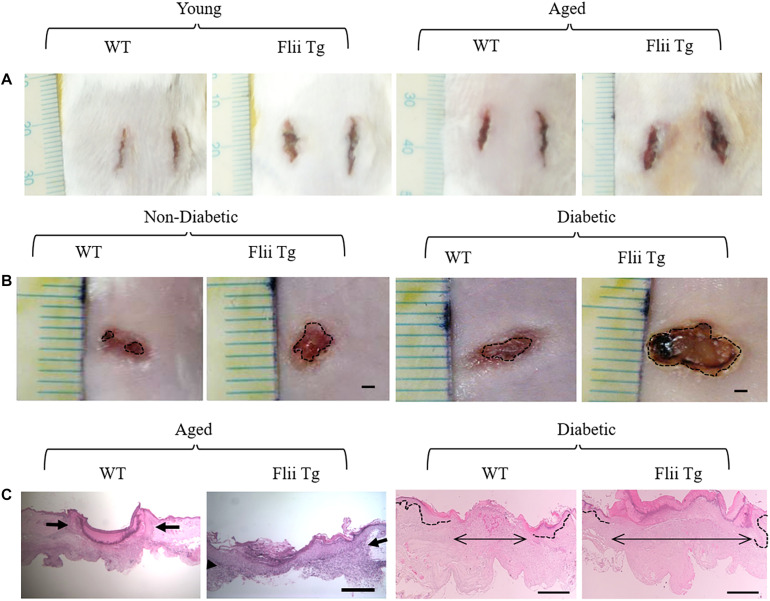
Flii impairs wound healing. **(A)** Incisional wound area at day 7 is increased in Flii overexpressing transgenic mice (Flii Tg) compared to wildtype (WT) in both young and aged mice. **(B)** Healing is also delayed in full thickness excisional wounds of both non-diabetic and diabetic mice with increased Flii with larger wound area on day 7 compared to WT mice. Histological analysis **(C)** confirmed that the distance between the dermal wound margins (arrows) was also larger in aged and diabetic Flii overexpressing wounds. Scale bar in B = 1 mm, C aged panels = 500 μm and C diabetic panels = 100 μm. Adapted with permission from Adams et al. (2008), Ruzehaji et al. (2013).

The levels of Flii are further increased in wounds with impaired healing, such as venous leg ulcers and diabetic foot wounds (Ruzehaji et al., 2013, 2014). Indeed, investigations in mice with altered Flii expression has shown that impaired healing associated with aging and diabetes is exacerbated by increased Flii expression (Figure 6; Adams et al., 2008; Ruzehaji et al., 2013). Flii appears to impair angiogenesis in diabetic wounds with endothelial cells isolated from Flii overexpressing mice showing disrupted tight junction formation and reduced micro vessel sprouting (Ruzehaji et al., 2014). Diabetic patients, which display elevated Flii expression, also have a reduced number of pericytes, which work in tandem with endothelial cells to form stable, functional blood vessels (Thomas et al., 2020). Heterozygous knockout of Flii results in an upregulation of pro-angiogenic VEGF expression, and increased numbers of both endothelial cells and pericytes in diabetic mouse wounds (Ruzehaji et al., 2014; Thomas et al., 2020). *In vitro*, treatment with FnAb stimulates HUVEC cells to form capillary tubes and FnAb-containing matrigel plugs inserted under the skin of mice were found to have a fourfold increase in the length of functional vessels that contained erythrocytes (Ruzehaji et al., 2014).

Flii is also increased in human burns and hypertrophic scars, with a similar increase seen in a mouse model of bleomycin-induced hypertrophic scaring (Cameron et al., 2016). The level of fibrosis caused by subcutaneous delivery of bleomycin in the skin reduces in Flii heterozygous knockout mice and have less TGF-β1 expression, but Flii overexpressing mice show increased scarring, higher numbers of myofibroblasts and an elevated collagen I/III ratio indicative of increased fibrosis (Cameron et al., 2016). TGFβ1 and β2 are both increased in the healing wounds of Flii overexpressing mice, whilst in Flii heterozygous knockout mice which heal faster, increased anti-scarring TGF-β3 isoform is observed (Chan et al., 2014). In blistered skin of EBA induced Flii heterozygous knockout mice, a reduced expression of contractile myofibroblast marker α-SMA is observed (Kopecki et al., 2011a). Reduced TGF-β1 and Smad2/3 expression is also observed within the blistered skin of the Flii heterozygous knockout mice (Kopecki et al., 2011a). Counterintuitively though, wounds of Flii heterozygous knockout mice appear more contracted and have significantly increased numbers of myofibroblasts compared to wild-type mice (Cowin et al., 2007). Whilst fibroblast-specific Flii overexpression impairs wound repair, with larger more gaping wounds at day 7 and reduced collagen I within the wounds, these same wounds have increased number of myofibroblasts (Turner et al., 2015).

It appears that the delayed wound closure observed in Flii overexpressing mice may be due in part to impairments in the process that prevents cellular migration over the provisional wound bed and through less strong adhesion at the dermal–epidermal junction (Kopecki et al., 2009). In wounds from Flii overexpressing mice decreased expression of Src, activated p130Cas and phosphorylated-paxillin is found (Kopecki et al., 2011b), indicating a direct link between this pathway and decreased wound closure observed in these mice. The expression of tetraspanin CD151, a key component of hemidesmosomes (Chometon et al., 2006) in the epidermis is also decreased in Flii overexpressing mice on day 3 and 7 post wounding (Kopecki et al., 2009). In addition to impacting the ability of cells to migrate into and repopulate the wound, Flii also affects the integrity of unwounded skin. The skin of Flii overexpressing mice is significantly thinner than WT, and has a reduced tensile strength (Kopecki et al., 2009). In fact, the formation of the epidermal barrier is delayed in embryos of mice that overexpress Flii and their adult counterparts exhibit increased intercellular space and trans epidermal water loss (Kopecki et al., 2014). Tight junction (TJ) formation appears to be impaired in these mice, with reduced expression of TJ proteins Claudin-1 and ZO-2 in Flii overexpressing embryos and altered localisation of these proteins in keratinocyte isolated from adult mice with increased Flii (Kopecki et al., 2014). Flii associates with TJ proteins, and while the exact mechanism by which Flii impairs epidermal barrier function is unknown, *in vivo* actin assays suggest that Flii inhibition of actin polymerization is of particular importance (Kopecki et al., 2014).

Wound healing investigations into the effect of LRRFIP1, with which Flii competes to influence the regulation of a number of pathways crucial to wound repair, may shed some light upon the specific Flii interactions that dictate the overall effect of Flii in a broader *in vivo* context. Kopecki et al. (2018b) report that LRRFIP1 is predominantly expressed by keratinocytes in unwounded skin, but upon wounding its expression is upregulated in both keratinocytes and fibroblasts. Addition of recombinant LRRFIP1 (rLRRFIP1) to human keratinocytes and fibroblasts *in vitro*, increases proliferation and metabolic activity (Kopecki et al., 2018b), similar to the effect of reducing Flii expression by siRNA knockdown and indicating that competition between Flii and LRRFIP1 upon the β-catenin-dependent proliferation may be a primary pathway affected. *In vivo*, the intradermal delivery of rLRRFIP1 to the margins of mouse incisional wounds exhibit similar effects to reduced Flii activity, with accelerated re-epithelialisation and smaller more contracted wounds by day 7 post-injury (Kopecki et al., 2018b). Interestingly, increased numbers of proliferating cells were only observed in the neoepidermis and not in the dermal wound fibroblasts (Kopecki et al., 2018b), whereas mice with reduced Flii exhibit increased proliferation in both (Cowin et al., 2007), suggesting that LRRFIP1 alone is not sufficient to prevent Flii inhibition of proliferation in wound fibroblasts. Similar to wounds with decreased Flii, rLRRFIP1 treated wounds contain decreased TLR4 expression, and a concomitant decrease in numbers of neutrophils and macrophages within the wound, and altered TGFβ1 and 3 expression, reminiscent of the effect of reducing Flii in wounds (Cowin et al., 2007; Adams et al., 2009; Ruzehaji et al., 2013; Kopecki et al., 2018b). No effect of increasing the levels of LRRFIP1 is observed upon angiogenesis (Kopecki et al., 2018b), suggesting that the positive effect upon angiogenesis seen by reducing Flii expression (Ruzehaji et al., 2014) is potentially independent of LRRFIP1.

As discussed earlier, Flii is a negative regulator of the immune response and *in vivo*, Flii expression peaks in mouse wounds around day 7 when resolution of the inflammatory stage of tissue repair is required (Cowin et al., 2007). In addition to being expressed by fibroblasts, keratinocytes and macrophages, Flii is also expressed by neutrophils within the blood and co-localizes with mature neutrophils within chronic wounds (Ruzehaji et al., 2012). Both NLRP3 KO and Caspase-1 KO mice have reduced inflammation (IL-1β, TNF-α, Neutrophils and Macrophages) at day 5 post excisional wounding, suggesting that reduced activity of the NLRP-3 inflammasome leads to an attenuated inflammatory response in wounds. Interestingly, this is also associated with delayed wound closure and reduced reepithelialisation, less granulation tissue formation and collagen deposition as well as impaired angiogenesis. Blocking caspase-1 on day 0 and 2 post wounding follows a similar trend (Weinheimer-Haus et al., 2015). Despite Flii overexpression being associated with delayed wound closure and reduced reepithelialisation, it is associated with increased granulation tissue formation and collagen deposition (Cowin et al., 2007) and indeed *in vivo* Flii appears to play a pro-inflammatory role. Increased MRP-14, a marker of immature macrophages and neutrophils is increased in the wounds of mice with fibroblast-specific Flii overexpression (Turner et al., 2015). In STZ-induced diabetic mice, overexpression of Flii leads to a concomitant increase in TLR4 expression and NF-B expression which is likely to further contribute to inflammation and chronicity (Ruzehaji et al., 2013). It has been suggested that the differences seen *in vivo* compared to cell based studies may not only be accounted for by the complexities of the wound environment, but by the timings in which responses are measured, that is *in vitro* measurements are taken within just a few hours, while the wound studies assess inflammation over a matter of days to a week (Ruzehaji et al., 2013). *In vivo* investigations using mice with altered Flii expression during the first few days of the inflammatory response may clarify the issue and articulate if initially Flii does indeed play an anti-inflammatory role.

### Flii Adversely Affects Chronic Inflammatory Conditions

The pro-inflammatory impacts of long term Flii overexpression have been investigated. In a mouse model of the chronic inflammatory skin disease, atopic dermatitis (AD), overexpression of Flii is associated with increased disease severity and tissue inflammation with higher TNFα and reduced IFN-γ expression (Kopecki et al., 2018a). While Flii heterozygous knockout mice exhibit reduced levels of inflammation and scaling, erythema and trans epidermal water loss and an overall more Th1 driven immune response during ovalbumin-induced AD, Flii overexpression leads to a TH2 skewed response and increased autoantibody reactivity (Kopecki et al., 2018a). In psoriasis, which is characterized by an excessive Th1 driven immune response and dysfunctional proliferation and differentiation of the epidermis, Flii expression is elevated throughout the epidermis, with higher expression in the differentiating upper spinous layer than in the proliferative basal layers of psoriatic skin (Chong et al., 2017). In psoriasiform, imiquimod-induced mice, Flii heterozygous knockout mice exhibit reduced skin thickening and inflammation, with proliferation within the epidermis restricted to the basal epidermis unlike WT and overexpressing mice in which proliferation is observed in the spinous layer (Chong et al., 2017). The expression of TLR4, which activates NF-κB to enhance inflammation during psoriasis, is reduced in Flii deficient mice with concomitant reductions in NF-κB and pro-inflammatory cytokine expression (Chong et al., 2017). Similarly, in the inflammatory bowel disease (IBD), Ulcerative Colitis, Flii levels are elevated (Kopecki et al., 2019). In a mouse model of IBD, reduced Flii expression in the Flii heterozygous knockout mice was associated with a decrease in disease severity and less shortening of the colon, whereas overexpression resulted in worsening of the disease compared to WT mice (Kopecki et al., 2019).

The dual role of Flii in regulating cellular inflammatory responses and stabilizing epidermal-dermal adherence increases its effect upon the autoimmune skin blistering disease Epidermolysis Bullosa Aquisita (EBA) (Kopecki et al., 2016). Flii expression is increased in the blistered skin of patients suffering the genetic skin blistering disorder Epidermolysis Bullosa (EB), in which patients exhibit extremely fragile skin (Kopecki et al., 2011a). Similarly, patients suffering from Kindler syndrome, which is also characterized by congenital blistering, exhibit elevated Flii levels (Kopecki et al., 2020). While this disorder arises from a loss of function of the Kindlin-1 integrin binding protein (Has et al., 2015), as Flii binds to this protein in keratinocytes and a reduction in Flii expression is associated with increased Kindlin-1 expression, it has been suggested that Flii may exacerbate blistering in these patients (Kopecki et al., 2020). Using a mouse model of acquired EBA, in which autoantibodies against collagen VII disrupt anchoring fibrils in the skin leading to sub-epidermal blisters, it was shown that Flii overexpression leads to more severe blistering, while blistering is reduced in Flii heterozygous knockout mice (Kopecki et al., 2011a). Flii overexpressing EBA mice show impaired TJ protein Claudin-1 and -4 expression with delayed barrier function recovery following blistering (Kopecki et al., 2014).

### Flii and Cancer Progression

Similar to wound healing, Flii appears to be both beneficial and detrimental in investigations of cancer progression in the skin (summarized in Table 2). Human Squamous Cell Carcinomas (SCC) display elevated levels of Flii compared to surrounding skin, particularly within invading cells at the tumor edge (Kopecki et al., 2015). The ability of the human SCC cell line (MET-1) to invade into a collagen/matrigel matrix can be reduced when Flii is reduced by treatment with a neutralising antibody (FnAb) *in vitro* (Kopecki et al., 2015). In an *in vivo* model of SCC, where cancerous lesions were induced by intradermal injection of 3-methylcholanthrene in mice with reduced, normal and elevated Flii expression, it was found that Flii overexpressing mice developed larger and more aggressive SCCs, whilst heterozygous knockout mice had significantly smaller, less invasive tumors (Kopecki et al., 2015). In particular, the tumors from mice with elevated Flii expression showed reduced caspase 1 expression and a concomitant reduction in the expression of apoptosis marker annexin V, suggesting that Flii contributes to SCC progression by decreasing apoptosis and increasing invasion by tumor cells (Kopecki et al., 2015).

**TABLE 2:** Flii and Cancer Prognosis.

Cancer Type	Flii Levels	Prognosis	References
**Negative Prognosis**			
Breast Cancer	High Akt-phosphorylated Flii Ser436	Poor patient prognosis Decreased survival time, increased tumor numbers and size High levels of Akt activity and p-Flii Ser436 impairs autophagic clearance and accumulation of insoluble proteins to progress breast cancer development.	He et al., 2018
Colorectal Cancer	High	Flii inhibits endoplasmic reticulum stress-induced apoptosis Larger tumor formation	Choi et al., 2020
Lung Carcinoma	Low	Increased invasion	Wang et al., 2017
Prostate Cancer	Low	Poorer patient prognosis	Wang et al., 2016
Squamous Cell Carcinoma	High	Poor patient prognosis Increased invasiveness Larger and more aggressive SCCs	Kopecki et al., 2015
**Positive Prognosis**			
Breast Cancer	Ulk-phosphorylated Flii Ser64	High levels of Ulk (and phospho-Ser64 Flii) is associated with improved clinical prognosis Prevents Akt-phospho-Ser436 Flii and accumulation of insoluble proteins	He et al., 2018
Prostate Cancer	High	Patients with high AR expression but with high Flii tumor expression experienced better overall survival Reintroduction of Flii sensitizes prostate cancer cells to chemotherapy	Wang et al., 2016

In breast cancer, Flii expression is much higher and increases with development of the disease, with high Flii expression associated with a poorer prognosis (He et al., 2018). In a mouse model of mammary cancer, while heterozygous knockout of Flii only slightly delayed the formation of tumors, it significantly increased the median survival time, with reduced tumor numbers and size (He et al., 2018). In breast cancer cells, Flii interacts with the selective autophagy receptor p62 which itself is overexpressed in breast cancer and is associate with poor patient prognosis (He et al., 2018). Upon induction of p62 with ubiquitinated proteins, Flii is phosphorylated by Akt at Ser436 and independent of its actin binding ability, recruits p62-associated cargoes to the Triton X-100 insoluble actin bundle fraction that prevents p62 from recognizing LC3,impeding autophagic clearance of ubiquitinated proteins within p62 cargoes (He et al., 2018). It is this accumulation of insoluble proteins that leads to breast cancer development in the presence of Ser436 phosphorylated Flii (He et al., 2018). High Flii levels are also found within colorectal tumor tissue, where is appears that Flii protects against endoplasmic reticulum stress induced apoptosis and results in larger tumor formation (Choi et al., 2020).

In breast cancer patients with high levels of Ulk1, a positive regulator of autophagy, clinical prognosis is improved (He et al., 2018). Like Akt, Ulk1 phosphorylates Flii, this time at Ser64, which inhibits the phosphorylation of Flii by Akt, preventing the recruitment of p62 cargoes to actin bundles and promotes autophagy (He et al., 2018). Indeed, monitoring Flii phosphorylation may be a useful biomarker of breast cancer prognosis as higher Ulk1 activity and p-Flii Ser64 correlates well with improved clinical outcomes in patients, while higher levels of Akt activity and p-Flii Ser436 negatively correlated with a good breast cancer prognosis (He et al., 2018).

The role of Flii in cancer progression is, however, not a straightforward one. An opposing effect of Flii upon invasion is evident in human bronchial epithelial cells, where Flii expression is reduced in lung carcinoma cells lines H1299 and A529 compared to normal cells and reducing Flii by siRNA knockdown stimulates invasion, whereas Flii overexpression shows inhibition of this process (Wang et al., 2017). While Flii expression is found to be reduced within prostate cancer lesions compared to adjacent normal tissue, patients with high levels of AR expression but whose tumors were found to express high levels of Flii experienced better overall survival (Wang et al., 2016). The AR is known to promote tumor progression in prostate cancer patients, particularly in patients which are no longer responsive to androgen deprivation therapy and suffer aggressive tumors, however, the detrimental effects of AR expression may be overcome by Flii, as overexpression of Flii reduces both tumor size and weight, and reintroduction of Flii to prostate cancer cells can sensitize the cells to chemotherapy drugs bicalutamide and enzalutamide (Wang et al., 2016).

One possible mechanism by which Flii impacts upon cancer cell invasion, but also epithelial migration during wound healing, may be through the regulation of epithelial–mesenchymal transition (EMT) which is a process strongly linked to carcinoma invasion (Kim et al., 2017). LRRFIP1 is a key regulator of EMT, and its repression inhibits migration and invasion in cancer cells, mediated by increased phosphorylation of β-catenin targeting it for destruction to reduce its nuclear localisation and decreasing the transcription of downstream EMT markers (Douchi et al., 2015). Moreover, silencing LRRFIP1 leads to increased expression of β-catenin and E-cadherin in the plasma membrane, leading to more stable adherens junctions and reduced migration and invasion (Douchi et al., 2015). It may be that Flii interplay with LRRFIP1 modulates both the expression of EMT markers and invasion ability in cancer cell, with further research required to confirm this possibility. Furthermore, Flii may in fact regulate the expression of numerous proteins involved in cancer regulation through its regulation of nuclear export and subsequent translation of mRNAs (Wang et al., 2017).

### Targeting Flii to Improve Healing Outcomes

A number of approaches for preventing the adverse effects of Flii on wound healing have been investigated, which have shown promise in improving healing outcomes. These primarily focus upon reducing the impact of Flii, either through the application of Flii neutralising antibodies (FnAb) that binds to extracellular Flii to reduce its local activity or alternatively delivering siRNA against Flii to reduce local levels of Flii within the wound. FnAb injected intradermally around incisional wound margins or at the edges of partial thickness scald burns in wild-type mice showed a significant improvement in the appearance of the wounds (Cowin et al., 2007; Adams et al., 2009). Levels of pro-scarring TGF-β1 protein were reduced while anti-scarring TGF-β3 was significantly elevated. Moreover, α-smooth muscle actin (α-SMA), a marker of contractile myofibroblasts in the developing scars was also reduced (Adams et al., 2009). Injecting FnAb into developing scars formed by bleomycin induction in mice also led to a significant reduction in the size of the scars and a reduction in the collagen I/III ratio (Cameron et al., 2016). Similarly, neutralizing Flii using FnAb in a large animal (porcine) model of excisional wound healing was found to improve the macroscopic appearance of early scars and increased the rate of reepithelialisation (Jackson et al., 2012). Intradermal FnAb improves diabetic healing in STZ-induced mice, increasing the expression of VEGF within the wounds (Ruzehaji et al., 2013, 2014) and the delivery of FnAb also resulted in a significant decrease in TLR4 expression but not NF-κB, suggesting alternate regulation of NFκB may be at play in these diabetic mice (Ruzehaji et al., 2013).

A number of cream formulations for the topical delivery of FnAb have been also been developed that allow for the prolonged and sustained release of FnAb into the epidermis and upper papillary dermis of porcine skin (Haidari et al., 2017). Topical application of FnAb cream to blistered skin in a mouse model of EBA reduces inflammatory cell infiltrate and when applied during the early stages of blister formation reduces blister severity (Kopecki et al., 2013). Likewise, treatment with FnAb cream reduces the severity of blisters when applied to established blisters, leading to stronger, less fragile skin (Kopecki et al., 2013). Similarly, topical application of FnAb prior to the induction of psoriasiform dermatitis in mice and continued application during the sensitisation of the skin, reduces skin inflammation and dermal cellular infiltration, leading to reduced erythema and epidermal hyperplasia (Chong et al., 2017).

In addition to neutralizing extracellular Flii with FnAb, a number of approaches to prevent the action of intracellular Flii *in vivo* have been investigated using small interfering RNA against Flii (Flii siRNA). In an attempt to decrease fibrotic processes associated with medical device implantation, Martens et al used a layer-by-layer polymer surface modification technique, alternating the deposition of poly-L-lysine and Flii siRNA to generate Rhodamine labeled-Flii siRNA coated implants for subcutaneous implantation in mice (Martens et al., 2015). After two days, cells that had adhered to the implants surface were found to be Rhodamine positive indicating cellular uptake of the Flii siRNA. Moreover, functionality of the siRNA was confirmed as Flii expression within the tissue surrounding the implants was decreased for up to seven days, with a concomitant reduction in TGF-β1 and increase in TGF-β3 demonstrating the potential utility of the methods to alter the fibrotic process (Martens et al., 2015).

Porous silicon nanoparticles (pSi NPs), which do not induce toxicity or inflammatory responses are broken down in the extracellular environment and upon degradation release their contents, have been used to deliver Flii into wounds where proteolytic degradation may reduce the efficacy of the antibody (Turner et al., 2017). *In vitro*, pSi NPs loaded with FnAb (FnAb-pSi NPs) were shown to release FnAb which retained its functionality to exert similar effects as FnAb treatment, increasing the recovery of keratinocytes wounded by electric cell-sensing impedance sensing and enhancing their proliferation (Turner et al., 2015). Mouse incisional wounds treated with a single dose of FnAb-pSi NPs at the time of injury, also had significantly smaller wound areas that those treated with unloaded pSi NPs (Turner et al., 2017). As acute wound environments do not have the same proteolytic environment as chronic wounds FnAb-pSi NPs were also administered to excisional wounds in diabetic mice (147). Following a single intradermal dose at the time of injury the diabetic wounds closed two days earlier than unloaded pSi NPs, and importantly, the FnAb-pSi NPs performed better than “naked” FnAb intradermal injections as the antibody was protected from proteolytic degradation (Turner et al., 2017).

## Summary and Conclusion

Ever since Flii was first discovered in 1993 as a gene responsible for muscle degeneration in the drosophila, a significant body of work has been undertaken which has identified broad-reaching functions of this actin-binding protein. As a member of the gelsolin family of actin remodeling proteins it is not unexpected that Flii has significant functions in regulating cellular processes including proliferation, adhesion, migration and apoptosis leading to potential important roles in pathological conditions that rely on the successful performance of these processes including wound healing and cancer. More surprisingly are the emerging roles of Flii in modulating signaling processes that affect inflammation and inflammatory conditions leading to the identification of Flii as a potential therapeutic target that may be important in the development of new approaches to treat different disease states. While much is known about the intracellular function of Flii its extracellular activities remain to be elucidated and may well form the next body of work that helps to explain the multifunctional and important roles of Flii.

## Author Contributions

XS prepared the original draft. AC did the review and editing. All authors contributed to the article and approved the submitted version.

## Conflict of Interest

AC is a shareholder in AbRegen Pty Ltd., which is developing antibodies against Flii for wound treatments. The remaining author declares that the research was conducted in the absence of any commercial or financial relationships that could be construed as a potential conflict of interest.

## References

[B1] Adams D. H. Ruzehaji N. Strudwick X. L. Greenwood J. E. Campbell H. D. Arkell R. (2009). Attenuation of Flightless I, an actin-remodelling protein, improves burn injury repair via modulation of transforming growth factor (TGF)-beta1 and TGF-beta3. *Br. J. Dermatol.* 161 326–336. 10.1111/j.1365-2133.2009.09296.x 19519830

[B2] Adams D. H. Strudwick X. L. Kopecki Z. Hooper-Jones J. A. Matthaei K. I. Campbell H. D. (2008). Gender specific effects on the actin-remodelling protein Flightless I and TGF-beta1 contribute to impaired wound healing in aged skin. *Int. J. Biochem. Cell Biol.* 40 1555–1569. 10.1016/j.biocel.2007.11.024 18191609

[B3] Akhter A. Caution K. Abu Khweek A. Tazi M. Abdulrahman B. A. Abdelaziz D. H. (2012). Caspase-11 promotes the fusion of phagosomes harboring pathogenic bacteria with lysosomes by modulating actin polymerization. *Immunity* 37 35–47. 10.1016/j.immuni.2012.05.001 22658523 PMC3408798

[B4] Alarcon-Sanchez B. R. Guerrero-Escalera D. Rosas-Madrigal S. Ivette Aparicio-Bautista D. Reyes-Gordillo K. Lakshman M. R. (2020). Nucleoredoxin interaction with flightless-I/actin complex is differentially altered in alcoholic liver disease. *Basic Clin. Pharmacol. Toxicol.* [Epub ahead of print]. 10.1111/bcpt.13451 32524749

[B5] Alexandrova A. Y. Arnold K. Schaub S. Vasiliev J. M. Meister J. J. Bershadsky A. D. (2008). Comparative dynamics of retrograde actin flow and focal adhesions: formation of nascent adhesions triggers transition from fast to slow flow. *PLoS One* 3:e3234. 10.1371/journal.pone.0003234 18800171 PMC2535565

[B6] Archer S. K. Behm C. A. Claudianos C. Campbell H. D. (2004). The flightless I protein and the gelsolin family in nuclear hormone receptor-mediated signalling. *Biochem. Soc. Trans.* 32 940–942. 10.1042/bst0320940 15506930

[B7] Archer S. K. Claudianos C. Campbell H. D. (2005). Evolution of the gelsolin family of actin-binding proteins as novel transcriptional coactivators. *Bioessays* 27 388–396. 10.1002/bies.20200 15770676

[B8] Arnaout M. A. Goodman S. L. Xiong J. P. (2007). Structure and mechanics of integrin-based cell adhesion. *Curr. Opin. Cell Biol.* 19 495–507. 10.1016/j.ceb.2007.08.002 17928215 PMC2443699

[B9] Arora P. D. Di Gregorio M. He P. Mcculloch C. A. (2017). TRPV4 mediates the Ca(2+) influx required for the interaction between flightless-1 and non-muscle myosin, and collagen remodeling. *J. Cell. Sci.* 130 2196–2208. 10.1242/jcs.201665 28526784

[B10] Arora P. D. He T. Ng K. Mcculloch C. A. (2018). The leucine-rich region of Flightless I interacts with R-ras to regulate cell extension formation. *Mol. Biol. Cell.* 29 2481–2493. 10.1091/mbc.e18-03-0147 30091651 PMC6233052

[B11] Arora P. D. Nakajima K. Nanda A. Plaha A. Wilde A. Sacks D. B. (2020). Flightless anchors IQGAP1 and R-ras to mediate cell extension formation and matrix remodeling. *Mol. Biol. Cell.* 31 1595–1610. 10.1091/mbc.e19-10-0554 32432944 PMC7521798

[B12] Arora P. D. Wang Y. Bresnick A. Janmey P. A. Mcculloch C. A. (2015). Flightless I interacts with NMMIIA to promote cell extension formation, which enables collagen remodeling. *Mol. Biol. Cell.* 26 2279–2297. 10.1091/mbc.e14-11-1536 25877872 PMC4462945

[B13] Bach C. T. Creed S. Zhong J. Mahmassani M. Schevzov G. Stehn J. (2009). Tropomyosin isoform expression regulates the transition of adhesions to determine cell speed and direction. *Mol. Cell. Biol.* 29 1506–1514. 10.1128/mcb.00857-08 19124607 PMC2648248

[B14] Bagashev A. Fitzgerald M. C. Larosa D. F. Rose P. P. Cherry S. Johnson A. C. (2010). Leucine-rich repeat (in Flightless I) interacting protein-1 regulates a rapid type I interferon response. *J. Interferon Cytokine Res.* 30 843–852. 10.1089/jir.2010.0017 20586614 PMC2992405

[B15] Bell J. K. Mullen G. E. Leifer C. A. Mazzoni A. Davies D. R. Segal D. M. (2003). Leucine-rich repeats and pathogen recognition in Toll-like receptors. *Trends Immunol.* 24 528–533. 10.1016/s1471-4906(03)00242-4 14552836

[B16] Bernad R. Engelsma D. Sanderson H. Pickersgill H. Fornerod M. (2006). Nup214-Nup88 nucleoporin subcomplex is required for CRM1-mediated 60 S preribosomal nuclear export. *J. Biol. Chem.* 281 19378–19386. 10.1074/jbc.m512585200 16675447

[B17] Berrier A. L. Yamada K. M. (2007). Cell-matrix adhesion. *J. Cell Physiol.* 213 565–573. 17680633 10.1002/jcp.21237

[B18] Biernacka A. Dobaczewski M. Frangogiannis N. G. (2011). TGF-β signaling in fibrosis. *Growth Factors* 29 196–202. 21740331 10.3109/08977194.2011.595714 PMC4408550

[B19] Burger D. Fickentscher C. De Moerloose P. Brandt K. J. (2016). F-actin dampens NLRP3 inflammasome activity via Flightless-I and LRRFIP2. *Sci. Rep.* 6:29834. 10.1038/srep29834 PMC4949445 27431477

[B20] Cameron A. M. Turner C. T. Adams D. H. Jackson J. E. Melville E. Arkell R. M. (2016). Flightless I is a key regulator of the fibroproliferative process in hypertrophic scarring and a target for a novel antiscarring therapy. *Br. J. Dermatol.* 174 786–794. 10.1111/bjd.14263 26521845

[B21] Campbell H. D. Fountain S. Mclennan I. S. Berven L. A. Crouch M. F. Davy D. A. (2002). Fliih, a gelsolin-related cytoskeletal regulator essential for early mammalian embryonic development. *Mol. Cell. Biol.* 22 3518–3526. 10.1128/mcb.22.10.3518-3526.2002 11971982 PMC133791

[B22] Campbell H. D. Fountain S. Young I. G. Claudianos C. Hoheisel J. D. Chen K. S. (1997). Genomic structure, evolution, and expression of human FLII, a gelsolin and leucine-rich-repeat family member: overlap with LLGL. *Genomics* 42 46–54. 10.1006/geno.1997.4709 9177775

[B23] Campbell H. D. Schimansky T. Claudianos C. Ozsarac N. Kasprzak A. B. Cotsell J. N. (1993). The *Drosophila melanogaster* flightless-I gene involved in gastrulation and muscle degeneration encodes gelsolin-like and leucine-rich repeat domains and is conserved in Caenorhabditis elegans and humans. *Proc. Natl. Acad. Sci. U.S.A.* 90 11386–11390. 10.1073/pnas.90.23.11386 8248259 PMC47987

[B24] Carpentier S. J. Ni M. Duggan J. M. James R. G. Cookson B. T. Hamerman J. A. (2019). The signaling adaptor BCAP inhibits NLRP3 and NLRC4 inflammasome activation in macrophages through interactions with Flightless-1. *Sci. Signal.* 12:eaau0615. 10.1126/scisignal.aau0615 31088976 PMC6604799

[B25] Chan H. Kopecki Z. Waters J. Powell B. C. Arkell R. Cowin A. J. (2014). Cytoskeletal protein Flightless I differentially affects TGF-β isoform expression in both in vitro and in vivo wound models. *Wound Pract. Res.* 22 169–181.

[B26] Chen K. S. Gunaratne P. H. Hoheisel J. D. Young I. G. Miklos G. L. Greenberg F. (1995). The human homologue of the *Drosophila melanogaster* flightless-I gene (flil) maps within the Smith-Magenis microdeletion critical region in 17p11.2. *Am. J. Hum. Genet.* 56 175–182. 7825574 PMC1801336

[B27] Cho I. K. Chang C. L. Li Q. X. (2013). Diet-induced over-expression of flightless-I protein and its relation to flightlessness in Mediterranean fruit fly, *Ceratitis capitata*. *PLoS One* 8:e81099. 10.1371/journal.pone.0081099 24312525 PMC3849048

[B28] Choe N. Kwon J. S. Kim J. R. Eom G. H. Kim Y. Nam K. I. (2013). The microRNA miR-132 targets Lrrfip1 to block vascular smooth muscle cell proliferation and neointimal hyperplasia. *Atherosclerosis* 229 348–355. 10.1016/j.atherosclerosis.2013.05.009 23880186

[B29] Choi C. K. Vicente-Manzanares M. Zareno J. Whitmore L. A. Mogilner A. Horwitz A. R. (2008). Actin and alpha-actinin orchestrate the assembly and maturation of nascent adhesions in a myosin II motor-independent manner. *Nat. Cell. Biol.* 10 1039–1050. 10.1038/ncb1763 19160484 PMC2827253

[B30] Choi J. S. Choi S. S. Kim E. S. Seo Y. K. Seo J. K. Kim E. K. (2015). Flightless-1, a novel transcriptional modulator of PPARgamma through competing with RXRalpha. *Cell. Signal.* 27 614–620. 10.1016/j.cellsig.2014.11.035 25479590

[B31] Choi S. S. Lee S. K. Kim J. K. Park H. K. Lee E. Jang J. (2020). Flightless-1 inhibits ER stress-induced apoptosis in colorectal cancer cells by regulating Ca(2+) homeostasis. *Exp. Mol. Med.* 52 940–950. 10.1038/s12276-020-0448-3 32504039 PMC7338537

[B32] Chometon G. Zhang Z. G. Rubinstein E. Boucheix C. Mauch C. Aumailley M. (2006). Dissociation of the complex between CD151 and laminin-binding integrins permits migration of epithelial cells. *Exp. Cell Res.* 312 983–995. 10.1016/j.yexcr.2005.12.034 16490193

[B33] Chong H. T. Yang G. N. Sidhu S. Ibbetson J. Kopecki Z. Cowin A. J. (2017). Reducing Flightless I expression decreases severity of psoriasis in an imiquimod-induced murine model of psoriasiform dermatitis. *Br. J. Dermatol.* 176 705–712. 10.1111/bjd.14842 27373931

[B34] Claudianos C. Campbell H. D. (1995). The novel flightless-I gene brings together two gene families, actin-binding proteins related to gelsolin and leucine-rich-repeat proteins involved in Ras signal transduction. *Mol. Biol. Evol.* 12 405–414. 7739382 10.1093/oxfordjournals.molbev.a040215

[B35] Cowin A. J. Adams D. H. Strudwick X. L. Chan H. Hooper J. A. Sander G. R. (2007). Flightless I deficiency enhances wound repair by increasing cell migration and proliferation. *J. Pathol.* 211 572–581. 10.1002/path.2143 17326236

[B36] Cowin A. J. Hatzirodos N. Teusner J. T. Belford D. A. (2003). Differential effect of wounding on actin and its associated proteins, paxillin and gelsolin, in fetal skin explants. *J. Investig. Dermatol.* 120 1118–1129. 10.1046/j.1523-1747.2003.12231.x 12787143

[B37] Cowin A. J. Lei N. Franken L. Ruzehaji N. Offenhauser C. Kopecki Z. (2012). Lysosomal secretion of Flightless I upon injury has the potential to alter inflammation. *Commun. Integr. Biol.* 5 546–549. 10.4161/cib.21928 23336022 PMC3541319

[B38] Critchley D. R. Holt M. R. Barry S. T. Priddle H. Hemmings L. Norman J. (1999). Integrin-mediated cell adhesion: the cytoskeletal connection. *Biochem. Soc. Symp.* 65 79–99. 10320934

[B39] Dai P. Jeong S. Y. Yu Y. Leng T. Wu W. Xie L. (2009). Modulation of TLR signaling by multiple MyD88-interacting partners including leucine-rich repeat Fli-I-interacting proteins. *J. Immunol.* 182 3450–3460. 10.4049/jimmunol.0802260 19265123

[B40] Davy D. A. Ball E. E. Matthaei K. I. Campbell H. D. Crouch M. F. (2000). The flightless I protein localizes to actin-based structures during embryonic development. *Immunol. Cell. Biol.* 78 423–429. 10.1046/j.1440-1711.2000.00926.x 10947868

[B41] Davy D. A. Campbell H. D. Fountain S. De Jong D. Crouch M. F. (2001). The flightless I protein colocalizes with actin- and microtubule-based structures in motile Swiss 3T3 fibroblasts: evidence for the involvement of PI 3-kinase and Ras-related small GTPases. *J. Cell. Sci.* 114 549–562. 11171324 10.1242/jcs.114.3.549

[B42] De Berker D. Wojnarowska F. Sviland L. Westgate G. E. Dawber R. P. Leigh I. M. (2000). Keratin expression in the normal nail unit: markers of regional differentiation. *Br. J. Dermatol.* 142 89–96. 10.1046/j.1365-2133.2000.03246.x 10651700

[B43] de Couet H. G. Fong K. S. Weeds A. G. Mclaughlin P. J. Miklos G. L. (1995). Molecular and mutational analysis of a gelsolin-family member encoded by the flightless I gene of *Drosophila melanogaster*. *Genetics* 141 1049–1059. 8582612 10.1093/genetics/141.3.1049 PMC1206829

[B44] Deng H. Xia D. Fang B. Zhang H. (2007). The Flightless I homolog, fli-1, regulates anterior/posterior polarity, asymmetric cell division and ovulation during *Caenorhabditis elegans* development. *Genetics* 177 847–860. 10.1534/genetics.107.078964 17720906 PMC2034648

[B45] Dogic D. Rousselle P. Aumailley M. (1998). Cell adhesion to laminin 1 or 5 induces isoform-specific clustering of integrins and other focal adhesion components. *J. Cell. Sci.* 111(Pt 6) 793–802. 9472007 10.1242/jcs.111.6.793

[B46] Douchi D. Ohtsuka H. Ariake K. Masuda K. Kawasaki S. Kawaguchi K. (2015). Silencing of LRRFIP1 reverses the epithelial–mesenchymal transition via inhibition of the Wnt/β-catenin signaling pathway. *Cancer Lett.* 365 132–140. 10.1016/j.canlet.2015.05.023 26047573

[B47] Easley C. A. T. Brown C. M. Horwitz A. F. Tombes R. M. (2008). CaMK-II promotes focal adhesion turnover and cell motility by inducing tyrosine dephosphorylation of FAK and paxillin. *Cell Motil. Cytoskeleton* 65 662–674. 10.1002/cm.20294 18613116 PMC2830206

[B48] Fong K. S. de Couet H. G. (1999). Novel proteins interacting with the leucine-rich repeat domain of human flightless-I identified by the yeast two-hybrid system. *Genomics* 58 146–157. 10.1006/geno.1999.5817 10366446

[B49] Funato Y. Michiue T. Asashima M. Miki H. (2006). The thioredoxin-related redox-regulating protein nucleoredoxin inhibits Wnt-beta-catenin signalling through dishevelled. *Nat. Cell. Biol.* 8 501–508. 10.1038/ncb1405 16604061

[B50] Gettemans J. Van Impe K. Delanote V. Hubert T. Vandekerckhove J. De Corte V. (2005). Nuclear actin-binding proteins as modulators of gene transcription. *Traffic* 6 847–857. 10.1111/j.1600-0854.2005.00326.x 16138899

[B51] Goetz J. G. (2009). Bidirectional control of the inner dynamics of focal adhesions promotes cell migration. *Cell. Adh. Migr.* 3 185–190. 10.4161/cam.3.2.7295 19398887 PMC2679883

[B52] Goodall A. H. Burns P. Salles I. Macaulay I. C. Jones C. I. Ardissino D. (2010). Transcription profiling in human platelets reveals LRRFIP1 as a novel protein regulating platelet function. *Blood* 116 4646–4656. 10.1182/blood-2010-04-280925 20833976 PMC2996120

[B53] Goshima M. Kariya K. Yamawaki-Kataoka Y. Okada T. Shibatohge M. Shima F. (1999). Characterization of a novel Ras-binding protein Ce-FLI-1 comprising leucine-rich repeats and gelsolin-like domains. *Biochem. Biophys. Res. Commun.* 257 111–116. 10.1006/bbrc.1999.0420 10092519

[B54] Haidari H. Zhang Q. Melville E. Kopecki Z. Song Y. Cowin A. J. (2017). Development of topical delivery systems for flightless neutralizing antibody. *J. Pharm. Sci.* 106 1795–1804. 10.1016/j.xphs.2017.03.012 28336300

[B55] Has C. Chmel N. Levati L. Neri I. Sonnenwald T. Pigors M. (2015). FERMT1 promoter mutations in patients with Kindler syndrome. *Clin. Genet.* 88 248–254. 25156791 10.1111/cge.12490

[B56] Hayashi T. Funato Y. Terabayashi T. Morinaka A. Sakamoto R. Ichise H. (2010). Nucleoredoxin negatively regulates Toll-like receptor 4 signaling via recruitment of flightless-I to myeloid differentiation primary response gene (88). *J. Biol. Chem.* 285 18586–18593. 10.1074/jbc.m110.106468 20400501 PMC2881784

[B57] He J. P. Hou P. P. Chen Q. T. Wang W. J. Sun X. Y. Yang P. B. (2018). Flightless-I blocks p62-mediated recognition of LC3 to impede selective autophagy and promote breast cancer progression. *Cancer Res.* 78 4853–4864. 10.1158/0008-5472.can-17-3835 29898994

[B58] Higashi T. Ikeda T. Murakami T. Shirakawa R. Kawato M. Okawa K. (2010). Flightless-I (Fli-I) regulates the actin assembly activity of diaphanous-related formins (DRFs) Daam1 and mDia1 in cooperation with active Rho GTPase. *J. Biol. Chem.* 285 16231–16238. 10.1074/jbc.m109.079236 20223827 PMC2871490

[B59] Hintermann E. Quaranta V. (2004). Epithelial cell motility on laminin-5: regulation by matrix assembly, proteolysis, integrins and erbB receptors. *Matrix Biol.* 23 75–85. 10.1016/j.matbio.2004.03.001 15246107

[B60] Huang T. Schor S. L. Hinck A. P. (2014). Biological activity differences between TGF-beta1 and TGF-beta3 correlate with differences in the rigidity and arrangement of their component monomers. *Biochemistry* 53 5737–5749. 10.1021/bi500647d 25153513 PMC4165442

[B61] Hutten S. Kehlenbach R. H. (2006). Nup214 is required for CRM1-dependent nuclear protein export in vivo. *Mol. Cell. Biol.* 26 6772–6785. 10.1128/mcb.00342-06 16943420 PMC1592874

[B62] Huveneers S. Danen E. H. (2009). Adhesion signaling - crosstalk between integrins, Src and Rho. *J. Cell. Sci.* 122 1059–1069. 10.1242/jcs.039446 19339545

[B63] Jackson J. E. Kopecki Z. Adams D. H. Cowin A. J. (2012). Flii neutralizing antibodies improve wound healing in porcine preclinical studies. *Wound Repair Regen.* 20 523–536. 22672080 10.1111/j.1524-475X.2012.00802.x

[B64] Jackson J. E. Kopecki Z. Anderson P. J. Cowin A. J. (2020a). In vitro analysis of the effect of Flightless I on murine tenocyte cellular functions. *J. Orthop. Surg. Res.* 15:170. 10.1186/s13018-020-01692-9 PMC7216515 32398080

[B65] Jackson J. E. Kopecki Z. Anderson P. J. Cowin A. J. (2020b). Increasing the level of cytoskeletal protein Flightless I reduces adhesion formation in a murine digital flexor tendon model. *J. Orthop. Surg. Res.* 15 362. 10.1186/s13018-020-01889-y PMC7450967 32854733

[B66] Jeong K. W. (2014). Flightless I (*Drosophila*) homolog facilitates chromatin accessibility of the estrogen receptor α target genes in MCF-7 breast cancer cells. *Biochem. Biophys. Res. Commun.* 446 608–613. 10.1016/j.bbrc.2014.03.011 24632205

[B67] Jeong K. W. Lee Y. H. Stallcup M. R. (2009). Recruitment of the SWI/SNF chromatin remodeling complex to steroid hormone-regulated promoters by nuclear receptor coactivator flightless-I. *J. Biol. Chem.* 284 29298–29309. 10.1074/jbc.m109.037010 19720835 PMC2785560

[B68] Jin H. L. Yang L. Jeong K. W. (2017). Flightless-I homolog regulates glucocorticoid receptor-mediated transcription via direct interaction of the leucine-rich repeat domain. *Mol. Biol. Rep.* 44 243–250. 10.1007/s11033-017-4106-3 28455686

[B69] Jin J. Yu Q. Han C. Hu X. Xu S. Wang Q. (2013). LRRFIP2 negatively regulates NLRP3 inflammasome activation in macrophages by promoting Flightless-I-mediated caspase-1 inhibition. *Nat. Commun.* 4:2075. 10.1038/ncomms3075 PMC3753543 23942110

[B70] Jones J. C. Hopkinson S. B. Goldfinger L. E. (1998). Structure and assembly of hemidesmosomes. *Bioessays* 20 488–494. 10.1002/(sici)1521-1878(199806)20:6<488::aid-bies7>3.0.co;2-i 9699461

[B71] Kim D. H. Xing T. Yang Z. Dudek R. Lu Q. Chen Y. H. (2017). Epithelial mesenchymal transition in embryonic development, tissue repair and cancer: a comprehensive overview. *J. Clin. Med.* 7:1. 10.3390/jcm7010001 29271928 PMC5791009

[B72] Kopecki Z. Arkell R. Powell B. C. Cowin A. J. (2009). Flightless I regulates hemidesmosome formation and integrin-mediated cellular adhesion and migration during wound repair. *J. Invest. Dermatol.* 129 2031–2045. 10.1038/jid.2008.461 19212345

[B73] Kopecki Z. Arkell R. M. Strudwick X. L. Hirose M. Ludwig R. J. Kern J. S. (2011a). Overexpression of the Flii gene increases dermal-epidermal blistering in an autoimmune ColVII mouse model of epidermolysis bullosa acquisita. *J. Pathol.* 225 401–413. 10.1002/path.2973 21984127

[B74] Kopecki Z. Has C. Yang G. Bruckner-Tuderman L. Cowin A. (2020). Flightless I, a contributing factor to skin blistering in Kindler syndrome patients? *J. Cutan. Pathol.* 47 186–189. 10.1111/cup.13597 31614010

[B75] Kopecki Z. Ludwig R. J. Cowin A. J. (2016). Cytoskeletal regulation of inflammation and its impact on skin blistering disease epidermolysis bullosa acquisita. *Int. J. Mol. Sci.* 17:1116. 10.3390/ijms17071116 27420054 PMC4964491

[B76] Kopecki Z. O’neill G. M. Arkell R. M. Cowin A. J. (2011b). Regulation of focal adhesions by flightless i involves inhibition of paxillin phosphorylation via a Rac1-dependent pathway. *J. Invest. Dermatol.* 131 1450–1459. 10.1038/jid.2011.69 21430700

[B77] Kopecki Z. Ruzehaji N. Turner C. Iwata H. Ludwig R. J. Zillikens D. (2013). Topically applied flightless I neutralizing antibodies improve healing of blistered skin in a murine model of epidermolysis bullosa acquisita. *J. Invest. Dermatol.* 133 1008–1016. 10.1038/jid.2012.457 23223144

[B78] Kopecki Z. Stevens N. E. Chong H. T. Yang G. N. Cowin A. J. (2018a). Flightless I alters the inflammatory response and autoantibody profile in an OVA-induced atopic dermatitis skin-like disease. *Front. Immunol.* 9:1833. 10.3389/fimmu.2018.01833 30147695 PMC6095979

[B79] Kopecki Z. Stevens N. E. Yang G. N. Melville E. Cowin A. J. (2018b). Recombinant leucine-rich repeat flightless-interacting protein-1 improves healing of acute wounds through its effects on proliferation inflammation and collagen deposition. *Int. J. Mol. Sci.* 19:2014. 10.3390/ijms19072014 29996558 PMC6073877

[B80] Kopecki Z. Yang G. Jackson J. Melville E. Calley M. Murrell D. (2015). Cytoskeletal protein Flightless I inhibits apoptosis, enhances tumor cell invasion and promotes cutaneous squamous cell carcinoma progression. *Oncotarget* 6 36426–36440. 10.18632/oncotarget.5536 26497552 PMC4742187

[B81] Kopecki Z. Yang G. Treloar S. Mashtoub S. Howarth G. S. Cummins A. G. (2019). Flightless I exacerbation of inflammatory responses contributes to increased colonic damage in a mouse model of dextran sulphate sodium-induced ulcerative colitis. *Sci. Rep.* 9:12792. 10.1038/s41598-019-49129-6 PMC6728368 31488864

[B82] Kopecki Z. Yang G. N. Arkell R. M. Jackson J. E. Melville E. Iwata H. (2014). Flightless I over-expression impairs skin barrier development, function and recovery following skin blistering. *J. Pathol.* 232 541–552. 10.1002/path.4323 24375017

[B83] Lazzaro M. A. Pepin D. Pescador N. Murphy B. D. Vanderhyden B. C. Picketts D. J. (2006). The imitation switch protein SNF2L regulates steroidogenic acute regulatory protein expression during terminal differentiation of ovarian granulosa cells. *Mol. Endocrinol.* 20 2406–2417. 10.1210/me.2005-0213 16740656

[B84] Lee Y. H. Campbell H. D. Stallcup M. R. (2004). Developmentally essential protein flightless I is a nuclear receptor coactivator with actin binding activity. *Mol. Cell. Biol.* 24 2103–2117. 10.1128/mcb.24.5.2103-2117.2004 14966289 PMC350567

[B85] Lee Y. H. Stallcup M. R. (2006). Interplay of Fli-I and FLAP1 for regulation of beta-catenin dependent transcription. *Nucleic Acids Res.* 34 5052–5059. 10.1093/nar/gkl652 16990252 PMC1636430

[B86] Lei N. Franken L. Ruzehaji N. Offenhauser C. Cowin A. J. Murray R. Z. (2012). Flightless, secreted through a late endosome/lysosome pathway, binds LPS and dampens cytokine secretion. *J. Cell. Sci.* 125 4288–4296. 10.1242/jcs.099507 22718342

[B87] Li J. Brieher W. M. Scimone M. L. Kang S. J. Zhu H. Yin H. (2007). Caspase-11 regulates cell migration by promoting Aip1-Cofilin-mediated actin depolymerization. *Nat. Cell. Biol.* 9 276–286. 10.1038/ncb1541 17293856

[B88] Li J. Yin H. L. Yuan J. (2008). Flightless-I regulates proinflammatory caspases by selectively modulating intracellular localization and caspase activity. *J. Cell. Biol.* 181 321–333. 10.1083/jcb.200711082 18411310 PMC2315678

[B89] Liao D. J. Thakur A. Wu J. Biliran H. Sarkar F. H. (2007). Perspectives on c-Myc, Cyclin D1, and their interaction in cancer formation, progression, and response to chemotherapy. *Crit. Rev. Oncog.* 13 93–158. 10.1615/critrevoncog.v13.i2.10 18197790

[B90] Liao S. Wang C. Tang D. Wei J. He Y. Xiong H. (2015). [Interaction of Flightless I with Nup88 and Importin beta]. *Sheng Wu Gong Cheng Xue Bao* 31 1247–1254. 26762046

[B91] Lim M.-S. Jeong K. W. (2014). Role of Flightless-I (*Drosophila*) homolog in the transcription activation of type I collagen gene mediated by transforming growth factor beta. *Biochem. Biophys. Res. Commun.* 454 393–398. 10.1016/j.bbrc.2014.10.100 25451260

[B92] Lin C.-H. Waters J. Powell B. Arkell R. Cowin A. (2011). Decreased expression of Flightless I, a gelsolin family member and developmental regulator, in early-gestation fetal wounds improves healing. *Mammal. Genome* 22 341–352. 10.1007/s00335-011-9320-z 21400204

[B93] Liu J. Bang A. G. Kintner C. Orth A. P. Chanda S. K. Ding S. (2005). Identification of the Wnt signaling activator leucine-rich repeat in Flightless interaction protein 2 by a genome-wide functional analysis. *Proc. Natl. Acad. Sci. U.S.A.* 102 1927–1932. 10.1073/pnas.0409472102 15677333 PMC548559

[B94] Liu M. Liu M. Li B. Zhou Y. Huang Y. Lan X. (2016). Polymorphisms of FLII implicate gene expressions and growth traits in Chinese cattle. *Mol. Cell. Probes* 30 266–272. 10.1016/j.mcp.2016.07.005 27453522

[B95] Liu Y. Zou Z. Zhu B. Hu Z. Zeng P. Wu L. (2015). LRRFIP1 inhibits hepatitis C virus replication by inducing type I interferon in hepatocytes. *Hepat. Mon.* 15:e28473. 10.5812/hepatmon.15(5)2015.28473 PMC4451274 26045710

[B96] Liu Y. T. Yin H. L. (1998). Identification of the binding partners for flightless I, A novel protein bridging the leucine-rich repeat and the gelsolin superfamilies. *J. Biol. Chem.* 273 7920–7927. 10.1074/jbc.273.14.7920 9525888

[B97] Lodish H. Berk A. Zipursky S. L. Matsudaira P. Baltimore D. Darnell J. (2000). “The dynamics of actin assembly,” in *Molecular Cell Biology*, 4th Edn, ed. Freeman W. H. (New York, NY: W. H. Freeman).

[B98] Lu J. Dentler W. L. Lundquist E. A. (2008). FLI-1 Flightless-1 and LET-60 Ras control germ line morphogenesis in *C. elegans*. *BMC Dev. Biol.* 8:54. 10.1186/1471-213X-8-54 18485202 PMC2396608

[B99] MacDonald B. T. Tamai K. He X. (2009). Wnt/beta-catenin signaling: components, mechanisms, and diseases. *Dev. Cell.* 17 9–26. 10.1016/j.devcel.2009.06.016 19619488 PMC2861485

[B100] Mangan M. S. J. Olhava E. J. Roush W. R. Seidel H. M. Glick G. D. Latz E. (2018). Targeting the NLRP3 inflammasome in inflammatory diseases. *Nat. Rev. Drug Discov.* 17:588. 10.1038/nrd.2018.97 30026524

[B101] Marei H. Carpy A. Woroniuk A. Vennin C. White G. Timpson P. (2016). Differential Rac1 signalling by guanine nucleotide exchange factors implicates FLII in regulating Rac1-driven cell migration. *Nat. Commun.* 7:10664. 10.1038/ncomms10664 PMC4759627 26887924

[B102] Martens P. J. Ly M. Adams D. H. Penzkover K. R. Strudwick X. Cowin A. J. (2015). In vivo delivery of functional Flightless I siRNA using layer-by-layer polymer surface modification. *J. Biomater. Appl.* 30 257–268. 10.1177/0885328215579422 25838352

[B103] Martin P. (1997). Wound healing–aiming for perfect skin regeneration. *Science* 276 75–81. 10.1126/science.276.5309.75 9082989

[B104] Mendoza M. C. Er E. E. Blenis J. (2011). The Ras-ERK and PI3K-mTOR pathways: cross-talk and compensation. *Trends Biochem. Sci.* 36 320–328. 10.1016/j.tibs.2011.03.006 21531565 PMC3112285

[B105] Miao E. A. Rajan J. V. Aderem A. (2011). Caspase-1-induced pyroptotic cell death. *Immunol. Rev.* 243 206–214. 10.1111/j.1600-065x.2011.01044.x 21884178 PMC3609431

[B106] Mogensen T. H. (2009). Pathogen recognition and inflammatory signaling in innate immune defenses. *Clin. Microbiol. Rev.* 22 240–273. 10.1128/cmr.00046-08 19366914 PMC2668232

[B107] Mohammad I. Arora P. D. Naghibzadeh Y. Wang Y. Li J. Mascarenhas W. (2012). Flightless I is a focal adhesion-associated actin-capping protein that regulates cell migration. *FASEB J.* 26 3260–3272. 10.1096/fj.11-202051 22581781

[B108] Moustakas A. Souchelnytskyi S. Heldin C. H. (2001). Smad regulation in TGF-beta signal transduction. *J. Cell. Sci.* 114 4359–4369. 11792802 10.1242/jcs.114.24.4359

[B109] Nag S. Larsson M. Robinson R. C. Burtnick L. D. (2013). Gelsolin: the tail of a molecular gymnast. *Cytoskeleton* 70 360–384. 10.1002/cm.21117 23749648

[B110] Nayak A. Reck A. Morsczeck C. Muller S. (2017). Flightless-I governs cell fate by recruiting the SUMO isopeptidase SENP3 to distinct HOX genes. *Epigenet. Chromatin* 10:15. 10.1186/s13072-017-0122-8 PMC5364561 28344658

[B111] Ohtsuka H. Oikawa M. Ariake K. Rikiyama T. Motoi F. Katayose Y. (2011). GC-binding factor 2 interacts with dishevelled and regulates Wnt signaling pathways in human carcinoma cell lines. *Int. J. Cancer* 129 1599–1610. 10.1002/ijc.25837 21140450

[B112] O’Leary H. Lasda E. Bayer K. U. (2006). CaMKIIbeta association with the actin cytoskeleton is regulated by alternative splicing. *Mol. Biol. Cell* 17 4656–4665. 10.1091/mbc.e06-03-0252 16928958 PMC1635389

[B113] Olson M. F. Marais R. (2000). Ras protein signalling. *Semin. Immunol.* 12 63–73. 10.1006/smim.2000.0208 10723799

[B114] Park J. E. Jang J. Lee E. J. Kim S. J. Yoo H. J. Lee S. (2018a). Potential involvement of *Drosophila* flightless-1 in carbohydrate metabolism. *BMB Rep.* 51 462–467. 10.5483/bmbrep.2018.51.9.153 30060781 PMC6177503

[B115] Park J. E. Lee E. J. Kim J. K. Song Y. Choi J. H. Kang M. J. (2018b). Flightless-I controls fat storage in *Drosophila*. *Mol. Cells* 41 603–611. 29890821 10.14348/molcells.2018.0120 PMC6030243

[B116] Pepin D. Paradis F. Perez-Iratxeta C. Picketts D. J. Vanderhyden B. C. (2013). The imitation switch ATPase Snf2l is required for superovulation and regulates Fgl2 in differentiating mouse granulosa cells. *Biol. Reprod.* 88:142. 10.1095/biolreprod.112.105742 23616592

[B117] Plikus M. V. Gay D. L. Treffeisen E. Wang A. Supapannachart R. J. Cotsarelis G. (2012). Epithelial stem cells and implications for wound repair. *Semin. Cell. Dev. Biol.* 23 946–953. 23085626 10.1016/j.semcdb.2012.10.001 PMC3518754

[B118] Ridley A. J. (2015). Rho GTPase signalling in cell migration. *Curr. Opin. Cell Biol.* 36 103–112. 10.1016/j.ceb.2015.08.005 26363959 PMC4728192

[B119] Rottner K. Hall A. Small J. V. (1999). Interplay between Rac and Rho in the control of substrate contact dynamics. *Curr. Biol.* 9 640–648. 10.1016/s0960-9822(99)80286-3 10375527

[B120] Ruzehaji N. Grose R. Krumbiegel D. Zola H. Dasari P. Wallace H. (2012). Cytoskeletal protein Flightless (Flii) is elevated in chronic and acute human wounds and wound fluid: neutralizing its activity in chronic but not acute wound fluid improves cellular proliferation. *Eur. J. Dermatol.* 22 740–750. 10.1684/ejd.2012.1878 23178274

[B121] Ruzehaji N. Kopecki Z. Melville E. Appleby S. L. Bonder C. S. Arkell R. M. (2014). Attenuation of flightless I improves wound healing and enhances angiogenesis in a murine model of type 1 diabetes. *Diabetologia* 57 402–412. 10.1007/s00125-013-3107-6 24292564

[B122] Ruzehaji N. Mills S. J. Melville E. Arkell R. Fitridge R. Cowin A. J. (2013). The influence of Flightless I on Toll-like-receptor-mediated inflammation in a murine model of diabetic wound healing. *Biomed. Res. Int.* 2013:389792. 10.1155/2013/389792 PMC3595111 23555084

[B123] Seward M. E. Easley C. A. T. Mcleod J. J. Myers A. L. Tombes R. M. (2008). Flightless-I, a gelsolin family member and transcriptional regulator, preferentially binds directly to activated cytosolic CaMK-II. *FEBS Lett.* 582 2489–2495. 10.1016/j.febslet.2008.06.037 18588881

[B124] Shamsi F. Xue R. Huang T. L. Lundh M. Liu Y. Leiria L. O. (2020). FGF6 and FGF9 regulate UCP1 expression independent of brown adipogenesis. *Nat. Commun.* 11:1421. 10.1038/s41467-020-15055-9 PMC7078224 32184391

[B125] Shrivastava A. Prasad A. Kuzontkoski P. M. Yu J. Groopman J. E. (2015). Slit2N inhibits transmission of HIV-1 from dendritic cells to T-cells by modulating novel cytoskeletal elements. *Sci. Rep.* 5:16833. 10.1038/srep16833 PMC4652184 26582347

[B126] Straub K. L. Stella M. C. Leptin M. (1996). The gelsolin-related flightless I protein is required for actin distribution during cellularisation in *Drosophila*. *J. Cell. Sci.* 109(Pt 1) 263–270. 8834811 10.1242/jcs.109.1.263

[B127] Strom A. C. Weis K. (2001). Importin-beta-like nuclear transport receptors. *Genome Biol.* 2:REVIEWS3008. 10.1186/gb-2001-2-6-reviews3008 PMC138946 11423015

[B128] Strudwick X. L. Waters J. M. Cowin A. J. (2017). Flightless I expression enhances murine claw regeneration following digit amputation. *J. Invest. Dermatol.* 137 228–236. 10.1016/j.jid.2016.08.019 27595936

[B129] Thomas H. M. Ahangar P. Hofma B. R. Strudwick X. L. Fitridge R. Mills S. J. (2020). Attenuation of flightless i increases human pericyte proliferation, migration and angiogenic functions and improves healing in murine diabetic wounds. *Int. J. Mol. Sci.* 21:5599. 10.3390/ijms21165599 32764293 PMC7460558

[B130] Truica C. I. Byers S. Gelmann E. P. (2000). Beta-catenin affects androgen receptor transcriptional activity and ligand specificity. *Cancer Res.* 60 4709–4713. 10987273

[B131] Turner C. T. Mcinnes S. J. Melville E. Cowin A. J. Voelcker N. H. (2017). Delivery of flightless i neutralizing antibody from porous silicon nanoparticles improves wound healing in diabetic mice. *Adv. Healthc. Mater.* 6:1600707. 10.1002/adhm.201600707 27869355

[B132] Turner C. T. Waters J. M. Jackson J. E. Arkell R. M. Cowin A. J. (2015). Fibroblast-specific upregulation of Flightless I impairs wound healing. *Exp. Dermatol.* 24 692–697. 10.1111/exd.12751 25959103

[B133] Wang C. Chen K. Liao S. Gu W. Lian X. Zhang J. (2017). The flightless I protein interacts with RNA-binding proteins and is involved in the genome-wide mRNA post-transcriptional regulation in lung carcinoma cells. *Int. J. Oncol.* 51 347–361. 10.3892/ijo.2017.3995 28498392

[B134] Wang T. Chuang T.-H. Ronni T. Gu S. Du Y.-C. Cai H. (2006). Flightless I homolog negatively modulates the TLR pathway. *J. Immunol.* 176 1355–1362. 10.4049/jimmunol.176.3.1355 16424162

[B135] Wang T. Gu S. Ronni T. Du Y. C. Chen X. (2005). In vivo dual-tagging proteomic approach in studying signaling pathways in immune response. *J. Proteome Res.* 4 941–949. 10.1021/pr050031z 15952741

[B136] Wang T. Song W. Chen Y. Chen R. Liu Z. Wu L. (2016). Flightless I homolog represses prostate cancer progression through targeting androgen receptor signaling. *Clin. Cancer Res.* 22 1531–1544. 10.1158/1078-0432.ccr-15-1632 26527749

[B137] Wang T. T. Phang J. M. (1995). Effects of estrogen on apoptotic pathways in human breast cancer cell line MCF-7. *Cancer Res.* 55 2487–2489. 7780952

[B138] Weinheimer-Haus E. M. Mirza R. E. Koh T. J. (2015). Nod-like receptor protein-3 inflammasome plays an important role during early stages of wound healing. *PLoS One* 10:e0119106. 10.1371/journal.pone.0119106 25793779 PMC4368510

[B139] Wilson S. A. Brown E. C. Kingsman A. J. Kingsman S. M. (1998). TRIP: a novel double stranded RNA binding protein which interacts with the leucine rich repeat of flightless I. *Nucleic Acids Res.* 26 3460–3467. 10.1093/nar/26.15.3460 9671805 PMC147727

[B140] Won Jeong K. Chodankar R. Purcell D. J. Bittencourt D. Stallcup M. R. (2012). Gene-specific patterns of coregulator requirements by estrogen receptor-α in breast cancer cells. *Mol. Endocrinol.* 26 955–966. 10.1210/me.2012-1066 22543272 PMC3355545

[B141] Wu L. Chen H. Zhu Y. Meng J. Li Y. Li M. (2013). Flightless I homolog negatively regulates ChREBP activity in cancer cells. *Int. J. Biochem. Cell. Biol.* 45 2688–2697. 10.1016/j.biocel.2013.09.004 24055811

[B142] Xu J. Liao L. Qin J. Xu J. Liu D. Songyang Z. (2009). Identification of Flightless-I as a substrate of the cytokine-independent survival kinase CISK. *J. Biol. Chem.* 284 14377–14385. 10.1074/jbc.m807770200 19293151 PMC2682886

[B143] Yang G. N. Strudwick X. L. Bonder C. Kopecki Z. Cowin A. J. (2020). Effect of flightless I expression on epidermal stem cell niche during wound repair. *Adv. Wound Care* 9 161–173. 10.1089/wound.2018.0884 32117580 PMC7047082

[B144] Yang L. Jeong K. W. (2019). Flightless-I mediates the repression of estrogen receptor alpha target gene expression by the glucocorticoid receptor in MCF-7 cells. *Endocr. J.* 66 65–74. 10.1507/endocrj.ej18-0343 30369516

[B145] Yang P. An H. Liu X. Wen M. Zheng Y. Rui Y. (2010). The cytosolic nucleic acid sensor LRRFIP1 mediates the production of type I interferon via a beta-catenin-dependent pathway. *Nat. Immunol.* 11 487–494. 10.1038/ni.1876 20453844

[B146] Zhang S. Qiu W. Chen Y.-G. Yuan F.-H. Li C.-Z. Yan H. (2015). Flightless-I (FliI) is a potential negative regulator of the Toll pathway in *Litopenaeus vannamei*. *Fish Shellfish Immunol.* 42 413–425. 10.1016/j.fsi.2014.10.023 25449702

[B147] Zhou Y. Sun J. Li C. Wang Y. Li L. Cai H. (2014). Characterization of transcriptional complexity during adipose tissue development in bovines of different ages and sexes. *PLoS One* 9:e101261. 10.1371/journal.pone.0101261 24983926 PMC4077742

